# PATZ1 (MAZR) Co-occupies Genomic Sites With p53 and Inhibits Liver Cancer Cell Proliferation via Regulating p27

**DOI:** 10.3389/fcell.2021.586150

**Published:** 2021-02-01

**Authors:** Zhen Long Ng, Jiamin Siew, Jia Li, Guanxu Ji, Min Huang, Xiaohua Liao, Sue Yu, Yuanyuan Chew, Chin Wen Png, Yongliang Zhang, Shijun Wen, Henry Yang, Yiting Zhou, Yun Chau Long, Zhi Hong Jiang, Qiang Wu

**Affiliations:** ^1^Department of Biochemistry, Yong Loo Lin School of Medicine, National University of Singapore, Singapore, Singapore; ^2^Cancer Science Institute of Singapore, Centre for Translational Medicine, Singapore, Singapore; ^3^State Key Laboratory of Quality Research in Chinese Medicines, Macau University of Science and Technology, Taipa, Macau; ^4^Department of Microbiology, Immunology Programme, Life Sciences Institute, Yong Loo Lin School of Medicine, National University of Singapore, Singapore, Singapore; ^5^Medicinal Chemistry and Molecular Medicine, State Key Laboratory of Oncology in South China, Sun Yat-sen University Cancer Center, Guangzhou, China; ^6^The Key Laboratory of Tissue Engineering and Regenerative Medicine of Zhejiang Province, Center for Stem Cell and Regenerative Medicine, Zhejiang University School of Medicine, Hangzhou, China

**Keywords:** PATZ1, liver cancer proliferation, CDKN1B/p27, p53, ChIP-seq

## Abstract

Liver cancer is the third most common cause of cancer death in the world. POZ/BTB and AT-hook-containing zinc finger protein 1 (PATZ1/MAZR) is a transcription factor associated with various cancers. However, the role of PATZ1 in cancer progression remains controversial largely due to lack of genome-wide studies. Here we report that PATZ1 regulates cell proliferation by directly regulating CDKN1B (p27) in hepatocellular carcinoma cells. Our PATZ1 ChIP-seq and gene expression microarray analyses revealed that PATZ1 is strongly related to cancer signatures and cellular proliferation. We further discovered that PATZ1 depletion led to an increased rate of colony formation, elevated Ki-67 expression and greater S phase entry. Importantly, the increased cancer cell proliferation was accompanied with suppressed expression of the cyclin-dependent kinase inhibitor CDKN1B. Consistently, we found that PATZ1 binds to the genomic loci flanking the transcriptional start site of *CDKN1B* and positively regulates its transcription. Notably, we demonstrated that PATZ1 is a p53 partner and p53 is essential for CDKN1B regulation. In conclusion, our study provides novel mechanistic insights into the inhibitory role of PATZ1 in liver cancer progression, thereby yielding a promising therapeutic intervention to alleviate tumor burden.

## Introduction

Hepatocellular carcinoma (HCC) is a primary malignant tumor that arises from the hepatocytes. Globally, HCC is the third leading cause of cancer mortality and fifth most common type of cancer (Parkin et al., 2005). Chronic infection with hepatitis B (HBV) and C viruses (HCV) is responsible for 75% of all primary HCC cases worldwide, with a higher incidence in developing countries (El-Serag, 2012). The pathogenesis of HCC arising from HBV and HCV infection begins with proliferation and apoptosis, followed by inflammation, fibrosis, cirrhosis and finally dysplasia (Ananthakrishnan et al., 2006). Chronic consumption of alcohol, another well-characterized risk factor of HCC, leads to the development of primary liver cancer via cirrhosis (Chacko and Samanta, 2016). HCC is also associated with non-alcoholic liver fatty disease (NAFLD), which is the hepatic manifestation of obesity and related metabolic disorders (Inayat et al., 2016). In NAFLD, accumulation of fat in the liver results in inflammation, leading to cirrhosis and eventually HCC (Chacko and Samanta, 2016). Cirrhosis is regarded as the most important risk factor for the development and progression of HCC regardless of its etiologies, with 90% of all HCC cases being accompanied by cirrhosis in the western hemisphere (El-Serag, 2011).

Despite the severity of HCC, there remain limited options of treatment for advanced stage patients (Kelley and Venook, 2013; Flores and Marrero, 2014). Recent studies have identified signaling pathways involved in the development of HCC. These include the Hippo-YAP pathway, the VEGFR/EGFR pathway, the Wnt/β-catenin pathway, the PI3K/AKT/mTOR pathway and the MAPK/ERK pathway (Ahn et al., 2013; Janku et al., 2014; Lanaya et al., 2014; Wang et al., 2014; Bai et al., 2015). Nevertheless, the prognosis of HCC needs to be improved and identification of more potential drug targets remains a high priority.

The POZ/BTB and AT-hook containing zinc finger protein 1 (PATZ1), also known as MAZR, ZNF278 and ZBTB19, is a transcription factor and a member of the POZ and Krüppel-like zinc finger (POK) protein family which are characterized by the presence of both the POZ/BTB domain and the C_2_H_2_ zinc finger motif (Lee and Maeda, 2012). PATZ1 regulates a plethora of cellular processes including spermatogenesis (Fedele et al., 2008); senescence (Cho et al., 2012); embryonic stem cell pluripotency and reprogramming (Ma et al., 2014a, b; Ow et al., 2014); T-cell development, as well as cancer development (Bilic et al., 2006; Sakaguchi et al., 2010; Abramova et al., 2013; Sakaguchi et al., 2015). In particular, PATZ1 functions as an oncogene in colon cancer by promoting cell cycle progression (Tian et al., 2008). However, on the contrary, PATZ1 has also been implicated as a tumor suppressor gene in lymphomas, thyroid and lung cancer (Chiappetta et al., 2015; Franco et al., 2016; Ho et al., 2016; Vitiello et al., 2016). Hence the role of PATZ1 as an oncogene or tumor suppressor has been proposed to depend on the cellular context and the presence of interacting proteins (Valentino et al., 2013; Keskin et al., 2015).

To date, the role of PATZ1 in HCC has not been investigated. We have systematically studied the molecular role of PATZ1 in liver cancer progression. We found that PATZ1 modulates liver cancer cell proliferation by regulating CDKN1B, a key cyclin-dependent kinase inhibitor.

## Materials and Methods

### Cell Culture

HepG2, Huh7 and Hep3B cells obtained from ATCC were maintained in DMEM/High Glucose (GE Healthcare) supplemented with 10% heat inactivated fetal bovine serum (GE Healthcare). Normal Human Primary Hepatocytes (human NHEPS Cells) obtained from Lonza were cultured according to the manufacturer’s instruction. Briefly, the human NHEPS^TM^ cells were cultured in the Hepatocyte Maintenance Medium (HMM Medium) and harvested within 12 h. All cells were incubated in 5% CO_2_ at 37°C.

### siRNA Transfection

*PATZ1* siRNA (sc-76072) and scrambled control siRNA (sc-37007) were purchased from Santa Cruz Biotechnology. Reverse transfections with siRNA were performed with Lipofectamine RNAiMax according to the manufacturer’s instructions.

### Total RNA Extraction and Quantitative Real-Time PCR (qRT-PCR)

RNA was extracted from cells with the TRIzol^®^ reagent. Reverse transcription was performed with the Superscript III First-Strand Synthesis System using oligo-dT primer (Invitrogen). Quantitative real time PCR was performed using Fast SYBR^®^ Green Master Mix (Bio-Rad Laboratories). The relative quantification of mRNA levels was computed using the 2^–ΔΔ*CT*^ method. Primer sequences used were listed in Supplementary Tables 1, 2.

### Gene Expression Microarray Profiling

Total RNA from siCTRL- and si*PATZ1*-treated HepG2 cells was extracted with the TRIzol^®^ reagent and purified with the RNeasy Mini Kit (Qiagen). cDNAs from reverse transcription were biotin-labeled prior to hybridization onto HumanHT-12 v4 Expression BeadChip (Illumina). Hierarchical clustering was performed with Cluster 3.0 on differentially expressed genes (Eisen et al., 1998). Raw fold change data was adjusted to center both genes and array by its mean prior to hierarchical clustering by the Euclidean distance similarity metric and average linkage. The heat-map was visualized with Java Treeview (Saldanha, 2004). Gene ontology analysis was performed using the Database for Annotation, Visualization and Integrated Discovery (DAVID) v6.8 (Huang et al., 2009b). The PATZ1 gene expression microarray data has been submitted to Gene Expression Omnibus (GEO) (the GEO number is GSE113859).

### Western Blot

Western blot was conducted as previously described (Lee et al., 2012). The following primary antibodies were used: anti-PATZ1 (sc-390577; Santa Cruz), anti-p53 (sc-126; Santa Cruz), anti-β-Actin (sc-47778; Santa Cruz) and anti-p27 (#3688; Cell Signaling).

### Chromatin Immunoprecipitation (ChIP) Assay, ChIP-qPCR and ChIP-Sequencing

Chromatin Immunoprecipitation was performed as described previously (Lee et al., 2012). Briefly, HepG2 cells were crosslinked with 37% formaldehyde for 10 min prior to neutralization with 0.2 M glycine for 5 min. Cell lysis and nuclear lysis were performed followed by sonication. ChIP was then performed with Dynabeads Protein G (Invitrogen) coated with anti-PATZ1 (sc-292109; Santa Cruz). For sequential ChIP (Re-ChIP), we used 20 mM DMP (dimethyl pimelimidate) to crosslink protein-protein interaction during the first ChIP (PATZ1 ChIP). The chromatin from the first ChIP was eluted by 0.13 ml elution buffer (10 mM Hepes pH 7.5, 1 mM EDTA, 1% BSA and 1% SDS). Subsequently the eluent was diluted into 1.1 ml FA 0.1% SDS buffer and was used as “chromatin” for the second ChIP (p53 ChIP). Enrichment fold of ChIP DNA was compared with the input DNA by performing qRT-PCR with the Fast SYBR^®^ Green Master Mix. For ChIP-seq, ChIP-DNA library was generated by Illumina ChIP Library Prep Kit (Illumina) from the PATZ1 ChIP-DNA. High-throughput sequencing was then performed with the HiSeq High Output v3 (Illumina). Genomic sequence reads were mapped to the GRCh37/hg19 human genome assembly. BWA-MEM was used to do the ChIP-seq mapping with human genome 19 assembly (hg19) (Li, 2014). After converting the PATZ1 mapped files to sorted bam format, heat maps of the binding reads in all human genes and the average reads distribution of the two factors were generated by ngsplot (Barrett et al., 2013; Shen et al., 2014). Peak analysis and annotation were performed by comparing the respective input file, and the binding peaks with 5–50 model fold and *q*-value < 0.05 were pointed out by MACS2 (Zhang et al., 2008). The location of the peaks was annotated with HOMER (Heinz et al., 2010). Binding motifs and consensus motifs of the inferred peaks were subjected to Discriminative Regular Expression Motif Elicitation (DREME)-ChIP in the MEME suite^1^. The PATZ1 ChIP-seq data has been submitted to Gene Expression Omnibus (GEO) (the GEO number is GSE114030).

### Cell Viability Assay

HepG2 cells were seeded at a density of 0.06 × 10^6^ cells per well in a 96-well plate prior to transfection with scrambled control or PATZ1 siRNA. Fresh medium containing 20 μL of CellTiter 96^®^ AQueous One Solution Reagent (Promega) was added for 2 h. Absorbance was read at 490 nm using the BioTek H1 Hybrid Plate Reader (BioTek).

### Colony Formation Assay

siCTRL- and si*PATZ1*-treated HepG2 cells were harvested and re-seeded at a density of 1,000 cells per well in a 6-well plate. The cells were stained with 0.2% crystal violet 7 days after re-seeding and photograph was taken. To quantify the intensity of the crystal violet stains, 1% SDS was used to solubilize the crystal violet stain followed by measuring the absorbance at 570 nm.

### Cell Cycle Analysis

Cell cycle analysis was performed with BrdU and 7AAD stained siCTRL- or si*PATZ1*-treated HepG2 cells subjected to flow cytometry. Cell staining with the FITC BrdU Flow kit (BD Biosciences) was performed according to the manufacturer’s instruction. Briefly, HepG2 cells were fixed and permeabilized followed by staining with BrdU and 7AAD. Flow cytometry was performed using the BD LSR Fortessa Flow Cytometry Analyser.

### Immunofluorescence

Immunofluorescence was carried out as previously described (Ma et al., 2014a). The antibodies used were anti-PATZ1 (sc-292109; Santa Cruz) and anti-Ki-67 (sc-15402; Santa Cruz).

### Luciferase Assay

Transient siRNA transfection of HepG2 cells was performed with scrambled control and PATZ1 siRNA for 48h. Luciferase reporter assay was carried out as previously described (Ow et al., 2014). The *CDKN1B*-luc construct containing the CDKN1B promoter region was used to determine the promoter activity. To construct the PATZ1-binding abrogated reporter plasmid, the putative PATZ1 binding site GGGGAGGTG (located at −31 to −23 from the transcriptional start site) was mutated to TTTTCAACA using Q5^®^ Site-Directed Mutagenesis Kit (NEB).

### Statistical Analysis

Numerical data were presented as mean ± SEM. Differences between groups were determined by Student’s *t*-test and significant differences were determined by *P* ≤ 0.05.

## Results

### PATZ1 Is Prevalent in Liver Cancer and Depletion of PATZ1 Increases Cell Cycle Transcriptional Signature

**FIGURE 1:**
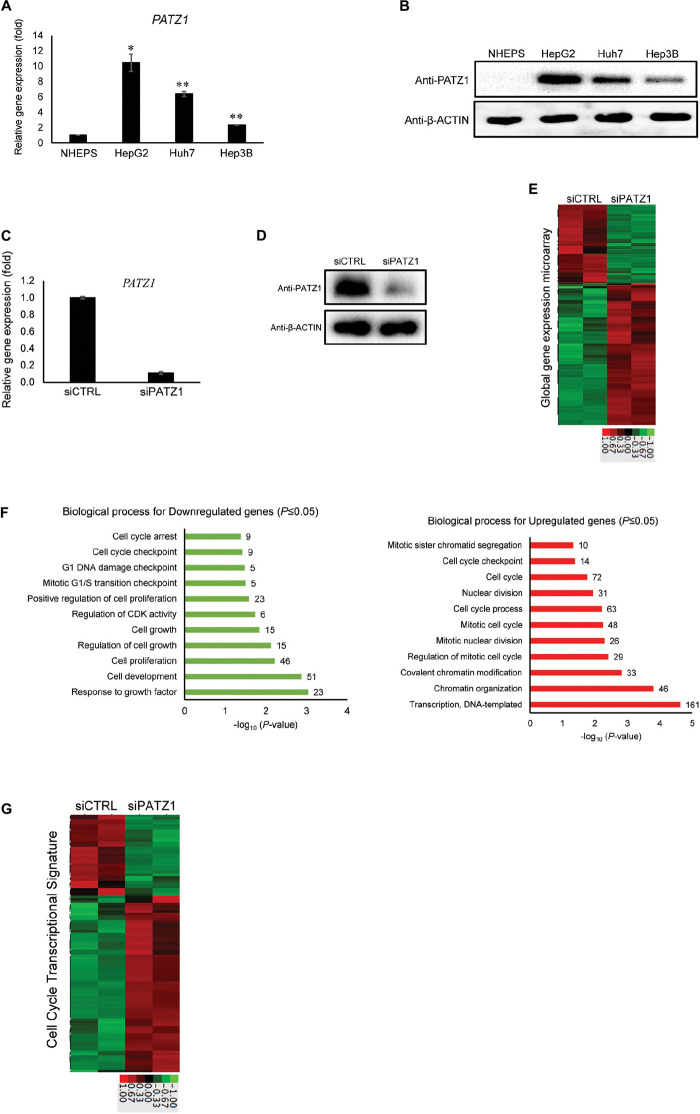
Enhanced global cell cycle transcriptome in PATZ1-deficient HepG2 cells. **(A)** PATZ1 expression level was evaluated in normal primary human hepatocytes (NHEPS) and HCC cell lines (HepG2, Huh7 and Hep3B). mRNA level of *PATZ1* was determined by qRT-PCR and normalized to the mRNA level of β-*ACTIN*. Data was presented as mean ± SEM; *n* = 2; *P ≤ 0.05, **P ≤ 0.01 (between NHEPS and the HCC cell lines via Student’s *t*-test). **(B)** Protein level of PATZ1 was determined by western blot. GAPDH was used as an equal loading control. **(C)** mRNA level of *PATZ1* in both siCTRL- and si*PATZ1*-treated HepG2 cells were determined by qRT-PCR and normalized to the mRNA level of β-*ACTIN*. Data was presented as mean ± SEM; *n* = 3; ***P ≤ 0.001 (between the groups treated with either siCTRL or si*PATZ1* via Student’s *t*-test). **(D)** Protein level of PATZ1 was determined by western blot. β-ACTIN was used as an equal loading control. **(E)** Duplicate gene expression microarray analysis of HepG2 cells treated with either siCTRL or si*PATZ1* were subjected to log_2_-transformed gene expression followed by *K*-means clustering. A fold-change threshold of 1.4 was applied. Data was represented in a thumbnail-dendrogram format. Upregulated and downregulated genes were indicated in red and green, respectively. The color scale bar below presents the fold change. **(F)** Differentially-expressed genes in si*PATZ1*-treated HepG2 cells were subjected to Gene Ontology (GO) clustering by DAVID. The bar chart depicts the biological processes of downregulated genes (left panel) and upregulated genes (right panel) of the PATZ1-deficient HepG2 transcriptome. Numerical values on the right indicate the number of genes associated with the respective biological processes. All GO groups demonstrated enhanced statistical representation (*P* ≤ 0.05). **(G)** Heatmap representing the transcriptome of siCTRL- and si*PATZ1*-treated HepG2 cells that is associated with the biological process “Cell cycle.” Upregulated and downregulated genes are indicated in red and green, respectively. The color scale bar below presents the fold change.

We examined mRNA and protein levels of PATZ1 in normal hepatocytes and liver cancer cell lines. We found that PATZ1 was expressed at higher levels in liver cancer cell lines HepG2, Huh7 and Hep3B compared to non-cancerous normal primary human hepatocytes NHEPS (Figures 1A,B). Publically available data from the Human Protein Atlas Project also show low to medium expression of PATZ1 in normal non-cancerous liver tissues compared to medium to high level in liver tumors (Uhlen et al., 2015). The overexpression of PATZ1 in liver cancer suggests a role of PATZ1 in liver cancer progression. As the HCC cell line HepG2 contained relatively higher level of PATZ1 compared to Huh7 and Hep3B, we used HepG2 to further investigate the role of PATZ1 in liver cancer.

To elucidate the relevance of PATZ1 overexpression in liver cancer, we first used siRNA to deplete PATZ1 level in HepG2 cells. Upon treatment with si*PATZ1*, the expression of PATZ1 was significantly reduced at both mRNA and protein levels (Figures 1C,D). Next, to investigate global gene expression changes caused by PATZ1 depletion, we performed gene expression microarray with the RNA extracted from PATZ1-depleted HepG2 cells (siCTRL-treated HepG2 cells as control). At a cut-off fold change of 1.4, we identified 447 downregulated genes and 795 upregulated genes in HepG2 cells treated with *PATZ1* siRNA (Figure 1E). Validation of the microarray data with qRT-PCR was performed on 11 arbitrary selected genes and the fold change results were generally in agreement (Supplementary Figure 1).

Next, we sought to identify biological processes affected by PATZ1 depletion. We carried out Gene Ontology (GO) clustering using DAVID (Huang et al., 2009a). Enriched GO terms among both downregulated and upregulated genes were related to cell cycle, including “Cell proliferation,” “Cell cycle process,” “Mitotic G1/S transition checkpoint,” and “Cell cycle checkpoint” (Figure 1F). Interestingly, enriched GO term among downregulated genes included “Regulation of cyclin-dependent kinase (CDK) activity” (Figure 1F, left panel). Specifically, *CDKN1A, CDKN1B* and *CDKN1C*, which belong to the Cip/Kip family of CDK inhibitors, were significantly downregulated upon *PATZ1* siRNA (Supplementary Figure 2A). The Cip/Kip family of CDK inhibitors restrict G_1_ to S phase progression of the cell cycle (Sherr and Roberts, 1999). Moreover, a heat-map of all differentially expressed genes annotated with the GO term “Cell cycle” show that these genes are mostly upregulated by *PATZ1* siRNA (Figure 1G). In total, expression of 105 cell-cycle related genes were affected by *PATZ1* knockdown. These genes account for 8.45% of all differentially expressed target genes in si*PATZ1*-treated HepG2 cells. KEGG pathway analysis further revealed an enrichment of pathways related to cell cycle and cancer among the differentially expressed target genes in the absence of PATZ1 (Supplementary Figure 2B). Together, these results suggest that PATZ1 is involved in the regulation of cancer-related genes, especially cell cycle genes, in HepG2 cells.

### PATZ1 Depletion Improves Cell Viability and Accelerates Cell Proliferation

**FIGURE 2:**
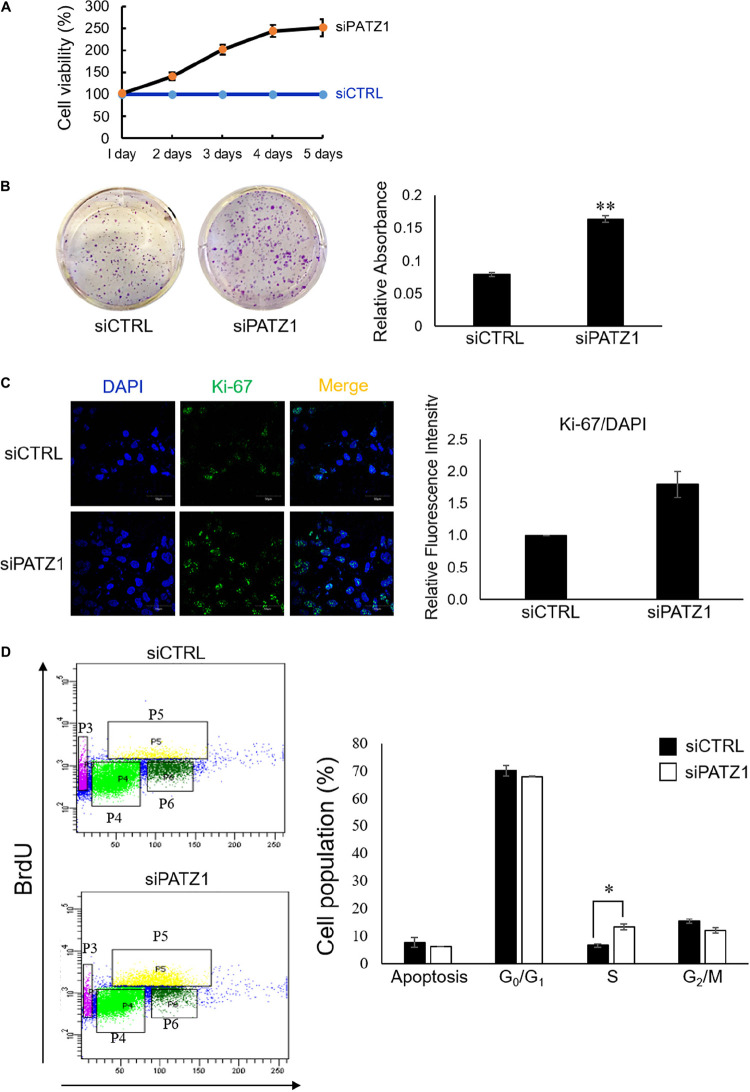
Deficiency of PATZ1 accelerates HepG2 proliferation. PATZ1 knockdown in HepG2 cells with either siCTRL or si*PATZ1* were conducted for the indicated time points. **(A)** Cell viability of HepG2 was determined by MTS assay at 1, 2, 3, 4, and 5 days after transfection. si*PATZ1*-treated HepG2 cells showed increased cell viability as compared to siCTRL-treated cells. **(B)** PATZ1 knockdown increases colony formation ability of HepG2 cells. Followed by treatment with either siCTRL- or si*PATZ1*-treated for 48 h, HepG2 cells were harvested and plated at a density of 1,000 cells and were subsequently stained with crystal violet after 7 days. **(C)** PATZ1-deficient HepG2 cells displayed increased Ki-67 protein levels. Ki-67 protein levels were quantified by immunofluorescence 48 h after siRNA-treatment of HepG2 cells. DAPI was used as a nuclear control. Relative fluorescence intensity of Ki-67/DAPI was determined by ImageJ (NIH). **(D)** Elevated incorporation of BrdU upon knockdown of PATZ1 in HepG2 cells. HepG2 cells were treated with either siCTRL- or si*PATZ1*-treated for 48 h prior to pulsing with 10 μM of BrdU for 1 h. The cells were then stained with anti-BrDU and 7AAD. Cell cycle analysis was performed with a flow cytometer. Cell populations belonging to different phases of the cell cycle were gated accordingly (P3: Apoptosis; P4: G_0_/G_1_; P5: S; P6: G_2_/M). Data was presented as mean ± SEM; *n* = 3; ^∗^*P* ≤ 0.05, ^∗∗^*P* ≤ 0.01 (between the groups treated with either siCTRL or si*PATZ1* via Student’s *t*-test).

To investigate the phenotypic effects of PATZ1 depletion, we first performed the MTS cell viability assay. We found that PATZ1 depletion raised cell viability in HepG2 cells in a time-dependent manner from 41% at 2 days, 102% at 3 days, 144% at 4 days and 151% at 5 days post-transfection (Figure 2A). The apparent increased cell viability in response to *PATZ1* knockdown could be due to an increase in cell proliferation. To test this, we assessed the colony forming ability of HepG2 upon PATZ1 depletion. Indeed, depletion of PATZ1 increased the rate of colony formation in si*PATZ1*-treated HepG2 cells as compared to siCTRL-treated cells (Figure 2B). This indicates PATZ1 may attenuate cell proliferation. To further elucidate the role of PATZ1 in cell proliferation, we immunostained si*PATZ1*-treated cells for Ki-67, a cell proliferation marker. Consistent with the colony formation assay, si*PATZ1*-treated cells showed twofold increase of Ki-67 expression level compared to siCTRL-treated cells (Figure 2C). Since cell proliferation is dependent on cell cycle progression, we next investigated the effect of PATZ1 depletion on percentages of cells in the different cell cycle phases. We discovered that *PATZ1* knockdown resulted in a twofold increase of BrdU-positive cells in the S phase (Figure 2D). Together these results demonstrate that PATZ1 depletion increases HepG2 cell proliferation by promoting S phase entry, leading to enhanced colony formation.

### Genome-Wide Mapping of PATZ1-Binding Sites

To gain molecular insights into the role of PATZ1 in regulating more cellular functions, we next identified binding sites of PATZ1 on a genome wide scale. To this end, we performed PATZ1 ChIP-seq in HepG2 cells. We determined putative binding sites of PATZ1 at PATZ1 ChIP-enriched genomic regions. We then validated these putative binding sites by performing ChIP-qPCR on arbitrarily selected genomic loci. The ChIP-qPCR validation at the cut-off value of fourfold matched an enrichment cut-off of sixfold as determined by the ChIP-seq analysis, which correspond to a total of 3,683 putative binding sites (Supplementary Figure 3).

**FIGURE 3:**
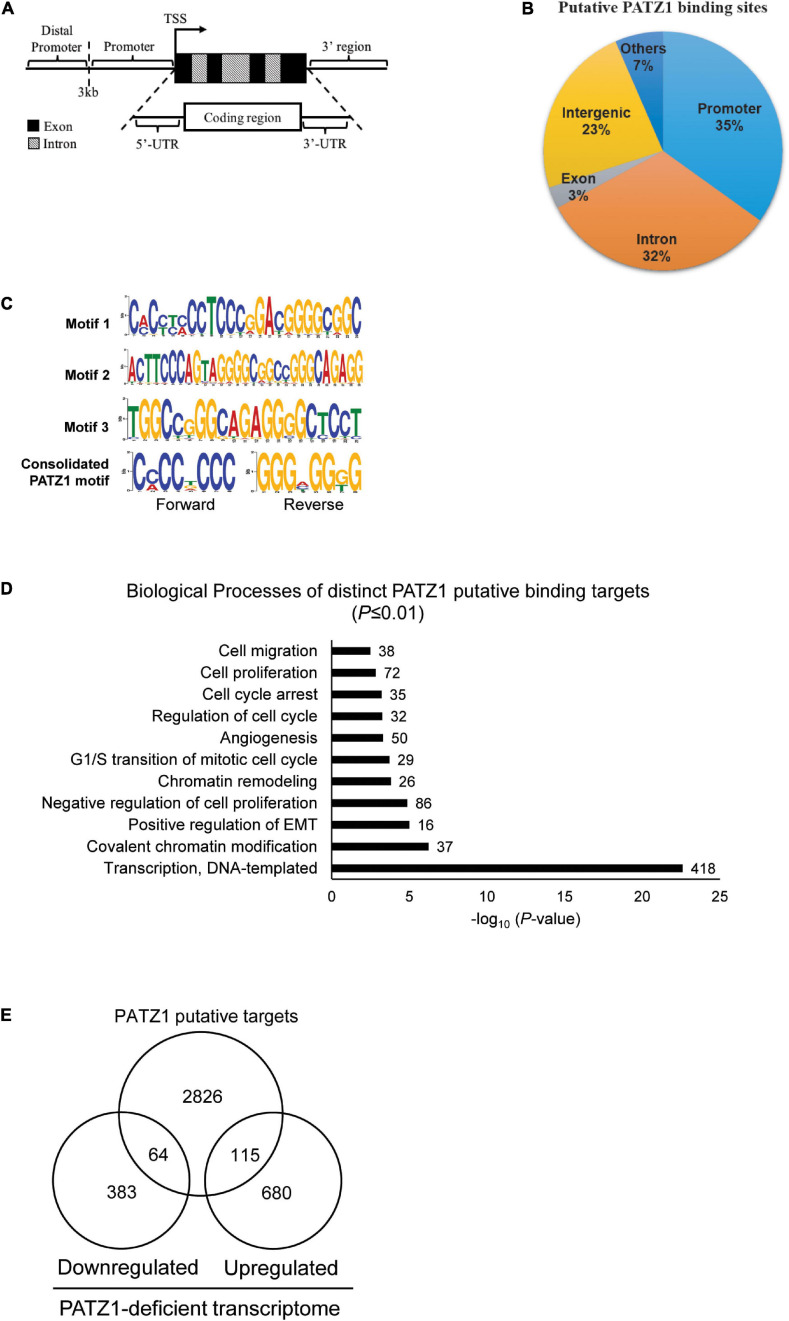
PATZ1 ChIP-sequencing analysis. **(A)** Illustration of the annotated genomic features employed to characterize the binding regions of PATZ1. **(B)** Genomic characterization of PATZ1 binding sites. **(C)** Computed putative binding motifs of PATZ1. Three motifs and a consensus motif were computationally determined from the PATZ1 ChIP-seq data. **(D)** Summary of the biological processes of PATZ1 ChIP-enriched targets which were subjected to GO clustering. All biological processes demonstrated enhanced statistical representation (All *P* ≤ 0.01). **(E)** Venn diagram depicting the overlap of PATZ1 ChIP-seq putative binding targets and genes whose expression was significantly altered in PATZ1 deficient HepG2 cells from the gene expression microarray. The ChIP-seq and gene expression microarray dataset was computed to reveal 64 potential genes that were downregulated and 115 potential genes that were upregulated directly by PATZ1.

We then annotated all binding sites to the nearest genes based on the GRCh37/hg19 genome assembly. We found that 35% of the putative PATZ1 binding sites lie within the promoters while 32% lie within the introns of the annotated genes (Figures 3A,B). Notably, 100% of the putative binding sites that lie within the promoter (<3 kb from the transcriptional start site; TSS) are within 1 kb from the TSS. On the other hand, 0.54% and 3.23% of the putative binding sites were located within the 3′-UTR and the 5′UTR, respectively.

Next, we looked for possible binding motifs of PATZ1 based on our ChIP-seq data. We found three PATZ1 binding motifs which were enriched in PATZ1 binding sites. Motif 1 is 24-nucleotides long and it primarily consists of both cytosine-rich (C-rich) and guanine-rich (G-rich) regions (Figure 3C, top panel). Motifs 2 and 3 are 29- and 21-nucleotides long, respectively, and both consist of G-rich region (Figure 3C, middle panel). Consolidation of the PATZ1 binding motifs results in a consensus motif of a C-rich region in the forward strand and a G-rich region in the reverse strand (Figure 3C, bottom panel), which is in good agreement with previous findings (Kobayashi et al., 2000; Ow et al., 2014). The high affinity of PATZ1 for G-rich sequences can be attributed to its zinc finger domains, which tends to bind to G-rich DNA (Jamieson et al., 1996).

We next sought to explore biological functions of genes bound by PATZ1. We identified 3,005 distinct annotated genes associated with 3,683 PATZ1 putative binding sites. GO analysis on these genes revealed strong enrichment in biological processes such as “G1/S transition of mitotic cell cycle,” “Regulation of cell cycle,” and “Cell proliferation” (Figure 3D). This is consistent with the GO clustering of our gene expression microarray results (Figure 1F). Remarkably, we found that 1624 (54%) of PATZ1 target genes are cancer-associated genes (Supplementary Figure 4), showing that PATZ1 target genes significantly overlap with oncogenic signature gene set (Molecular Signatures Database) (Esposito et al., 2011). Taken together, we suggest that PATZ1 regulates many cancer-related genes. Our Integrative ChIP-seq and microarray analyses revealed 64 target genes were significantly downregulated and 115 genes were upregulated upon PATZ1 depletion (Figure 3E).

### PATZ1 Binds to the TSS of *CDKN1B* and Regulates *CDKN1B* Transcription

**FIGURE 4:**
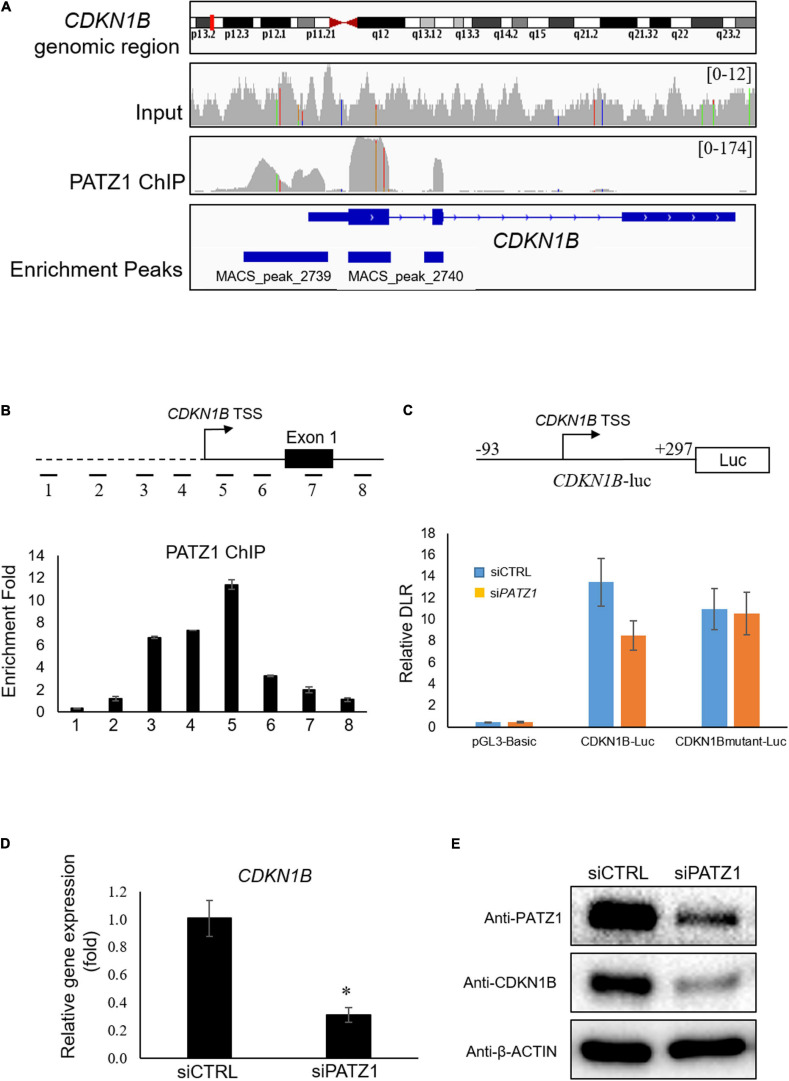
PATZ1 positively regulates CDKN1B by binding to its proximal promoter region. **(A)** Snapshot of the PATZ1 ChIP-seq data in HepG2 cells as visualized in the Integrative Genomic Viewer (Broad Institute). The top-most panel indicates the CDKN1B genomic region that was being interrogated. The second and third panels indicate the enrichment peaks of either the input DNA or the PATZ1 ChIP DNA for the corresponding genomic region of CDKN1B. The peak height o for the PATZ1 ChIP DNA depicts the binding region of PATZ1 to the genomic loci of CDKN1B with strong enrichment at the proximal promoter, exons 1 and 2 as indicated by the enrichment peaks in the bottom panel. **(B)** Locations of real-time PCR primers (amplicons 1 to 8) were mapped to the promoter regions of CDKN1B promoter. ChIP-qPCR was performed with PATZ1 ChIP-DNA. Strong enrichment of PATZ1 to the region TSS +9/+168 of the CDKN1B gene. **(C)** Luciferase reporter assay of CDKN1B promoter. Upper: schematic diagram of the luciferase vector *CDKN1B-luc*. The PATZ1 binding region (and its mutant) at CDKN1B promoter was cloned into the pGL3-Basic luciferase vector. Luciferase reporter assay was performed and normalized against the renilla signal to obtain the relative dual luciferase ratio (DLR), which represents the activity of the CDKN1B promoter region. **(D)** CDKN1B expression level was evaluated in siCTRL- and siPATZ1-treated HepG2 cells. Level of CDKN1B was normalized to mRNA level of β-ACTIN. Data was presented as mean ± SEM; *n* = 3; *P ≤ 0.05 via Student’s *t*-test. **(E)** Protein level of CDKN1B was determined by western blot. β-ACTIN was used as loading control.

Interestingly, we found that *CDKN1B* gene is bound by PATZ1 as indicated by the Model-based Analysis of ChIP-seq (MACS). PATZ1 ChIP-seq results showed that PATZ1 bound to the promoter, exon 1 and exon 2 of *CDKN1B* gene (Figure 4A). Indeed, *CDKN1B* was found to be among the top 10 targets of PATZ1 according to ChIP-seq enrichment (Supplementary Table 3). Furthermore, *CDKN1B* was significantly downregulated in PATZ1-deficient cells from the gene expression microarray analysis (Supplementary Figure 2). Since CDKN1B is important in cell cycle regulation, we propose that PATZ1 mediates the regulation of CDKN1B in cell cycle progression.

To validate the binding of PATZ1 to genomic sites of *CDKN1B*, we used ChIP-qPCR. Eight amplicons in genomic regions flanking the TSS of *CDKN1B* were selected (Figure 4B, top). We found that amplicon 5 corresponding to the region TSS +9/+168 was highly enriched in PATZ1 ChIP-DNA by 11.4 fold compared to a control region (Figure 4B, bottom). Amplicons 3 and 4 were also significantly enriched, indicating binding of PATZ1 to the immediate regions flanking the TSS of *CDKN1B*. ChIP-qPCR against amplicons 1 to 8 in HCT116 colon cancer cell line, H1 human embryonic stem cell (hESC) and HCC cell line Hep3B also revealed strong enrichment of PATZ1 to amplicon 5, further supporting binding of PATZ1 to the *CDKN1B* gene (Supplementary Figures 5A–D). Indeed, a putative PATZ1 binding site GGGGAGGTG (located at −31 to −23 from transcriptional start site) was found in the region which is close to amplicons 4 and 5 (Supplementary Figure 5E).

Next, to determine if binding of PATZ1 to *CDKN1B* results in transcriptional regulation, we performed luciferase reporter assay. The *CDKN1B* genomic region from −93 to +297 (*CDKN1B*-luc), which corresponds to amplicon 5 and its flanking genomic region, was tested (Figure 4C, top panel). As expected, *PATZ1* knockdown significantly reduced the promoter activity of this promoter fragment by approximately 33% while the promoter activity was insignificantly affected by depletion of PATZ1 if the putative PATZ1 binding site GGGGAGGTG (located at −31 to −23 from transcriptional start site) was mutated to TTTTCAACA (Figure 4C, bottom panel). This suggests that PATZ1 positively regulates the transcription of *CDKN1B*. Subsequently, we found that si*PATZ1*-treated HepG2 cells possessed reduced mRNA and lowered protein expression of CDKN1B compared to siCTRL-treated cells (Figures 4D,E). Taken together, these results confirm that PATZ1 positively regulates the transcription of *CDKN1B*.

### Regulation of CDKN1B by PATZ1 Is Dependent on p53

**FIGURE 5:**
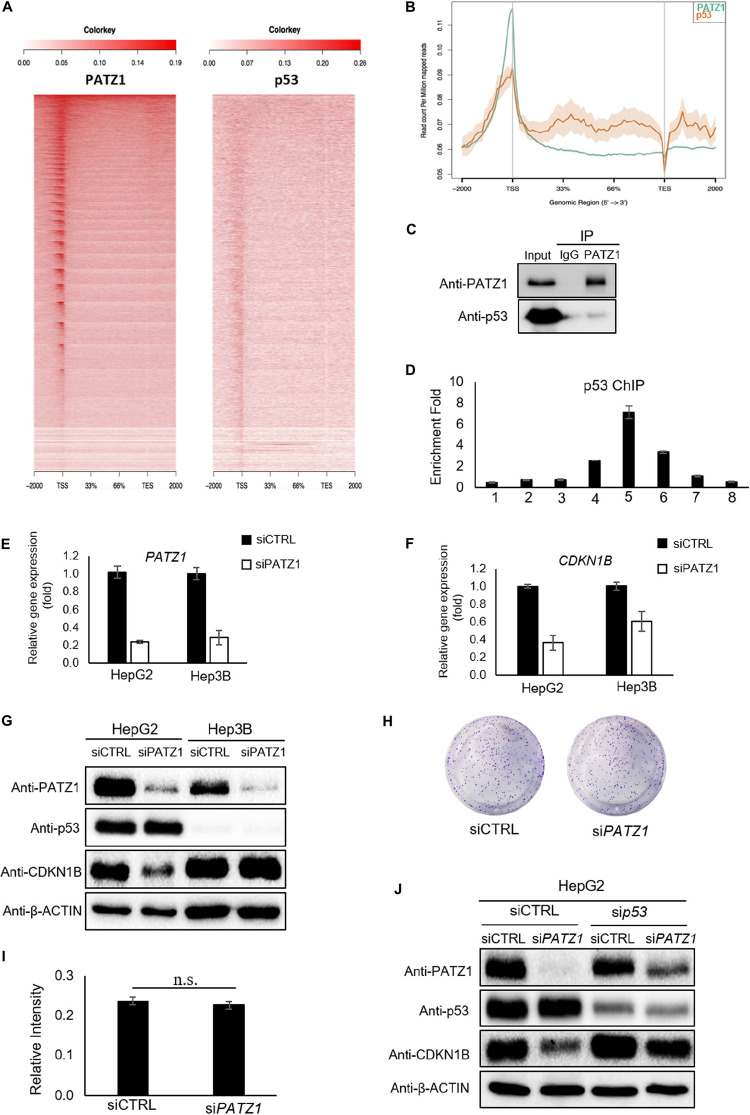
Regulation of CDKN1B by PATZ1 Is Dependent on p53. **(A)** Snapshot of the PATZ1 ChIP-seq data in HepG2 cells as visualized in the Integrative Genomic Viewer (Broad Institute). ngsplot was applied to delineate the relation between PATZ1 and p53. Heatmap depicting the distribution of the average reads of both PATZ1 and p53 ChIP results. **(B)** Consolidation of the number of peaks mapped to its respective genomic location. **(C)** Endogenous immunoprecipitation revealed interaction between PATZ1 and p53 in HepG2 cells. **(D)** ChIP-qPCR results indicated a strong enrichment of p53 to the promoter region (amplicon 5) of CDKN1B in HepG2 cells. Amplicons 1 to 8 depict the genomic loci of CDKN1B being probed in the ChIP-qPCR. **(E)** mRNA expression of PATZ1 in siCTRL and siPATZ1 HepG2 and Hep3B cells were determined by qRT-PCR. β-ACTIN acts as an endogenous control. **(F)** mRNA expression of CDKN1B in siCTRL and siPATZ1 HepG2 and Hep3B cells were determined by qRT-PCR. PATZ1-deficient HepG2 and Hep3B cells revealed declined CDKN1B expression. **(G)** Downregulation of PATZ1 does not affect CDKN1B protein expression in the p53-null Hep3B cells as compared to HepG2 cells. PATZ1, p53 and CDKN1B activity were determined by western blot analysis. β-ACTIN was used as a loading control. **(H)** Insignificant changes in colony formation in siCTRL- and siPATZ1-treated Hep3B cells shown by crystal violet stain. **(I)** Insignificant changes in colony formation in siCTRL- and siPATZ1-treated Hep3B cells shown by 570 nm absorbance. **(J)** siRNA-mediated p53 knockdown (sip53) in HepG2 cells abrogated the downregulation of CDKN1B in PATZ1-depleted cells as compared to the control. PATZ1, p53, and CDKN1B activity were determined by western blot analysis. β-ACTIN was used as a loading control. The numeric value was presented as mean ± S.E. Student’s *t*-tests were completed and statistical significance is indicated.

Previous studies have demonstrated an altered function of PATZ1 in response to the presence or absence of p53 (Valentino et al., 2013; Keskin et al., 2015). Therefore, to further investigate if PATZ1 cooperates with p53 and co-occupies genomics sites in liver cancer cells, we performed comparative ChIP-Seq analysis with the PATZ1 ChIP-Seq data in this study together with a publically available HepG2 p53 ChIP-Seq data (GEO Accession: GSM1581946). Notably, we observed both PATZ1 and p53 were found to bind to similar genomic regions at the TSS shown by the ngsplot heat-map (Figure 5A). Moreover, the binding intensities of PATZ1 and p53 to the TSS are concordant with one another, where the binding propensity of p53 reduces with the declining binding intensity of PATZ1. A graphical representation of the binding peaks of PATZ1 and p53 also revealed similar binding loci at the TSS region (Figure 5B). These suggest that PATZ1 is a p53 partner and co-regulates many downstream genes together with p53 in liver cancer cells.

We further found that endogenous PATZ1 physically interacts with p53 in HepG2 cells (Figure 5C), consistent with previous studies which showed interaction between PATZ1 and p53 in HEK293T and HCT116 cells (Valentino et al., 2013; Keskin et al., 2015). Given that PATZ1 binds to the *CDKN1B* genomic region in HepG2 cells, we next examined the presence of p53 in regions flanking the TSS of *CDKN1B*. Interestingly, we found two putative p53 binding sites at *CDKN1B* promoter region (Supplementary Figure 5E). p53 ChIP-qPCR revealed an enrichment of 7-folds of p53 to amplicon 5 (Figure 5D). Notably, the enrichment peak of p53 ChIP-qPCR corresponded to the enrichment peaks of PATZ1 ChIP-qPCR against the *CDKN1B* genomic loci. To confirm PATZ1 and p53 co-occupy this *CDKN1B* promoter region, we carried out sequential ChIP (1st ChIP, PATZ1; 2nd ChIP, p53). Our qPCR results clearly showed that the sequential ChIP largely enhanced the enrichment fold, showing that PATZ1 may cooperate with p53 in regulating *CDKN1B* transcription (Supplementary Figure 5F). Furthermore, comparative PATZ1 and a publically available p53 ChIP-seq analysis revealed that the bulk of the p53 binding loci coincide with that of the PATZ1 binding loci, which lies primarily at the TSS (Figure 5A). This observation strongly supports the cooperation of PATZ1 and p53 in regulation of CDKN1B.

Thus we further investigated the role of PATZ1 in regulating CDKN1B in a p53-null hepatocellular carcinoma cell line, Hep3B (Supplementary Figure 6). To evaluate the importance of p53 in the regulation of CDKN1B by PATZ1, siRNA-mediated knockdown of PATZ1 was performed in HepG2 and Hep3B cells (Figure 5E). With respect to the control cells, we observed that CDKN1B mRNA level was reduced by 64% (*P* ≤ 0.05) and 39% (*P* ≥ 0.05) in PATZ1-deficient HepG2 cells and Hep3B cells, respectively (Figure 5F). Interestingly, CDKN1B protein level remained unaltered in Hep3B cells upon PATZ1 knockdown (Figure 5G). Phenotypically, there was no significant change in colony forming ability between siCTRL- and siPATZ1-treated Hep3B cells (Figures 5H,I). Similarly, p53 knockdown in HepG2 cells abrogated the downregulation of CDKN1B upon PATZ1 depletion (Figure 5J).

Together, this showed that the absence of p53 not only abrogated the PATZ1-mediated regulation of CDKN1B, it nullified the ability of PATZ1 to phenotypically affect the colony forming ability of liver cancer cells. Taken together, these results indicated that PATZ1 partners with p53 and p53 is required for the regulation of CDKN1B by PATZ1.

**FIGURE 6:**
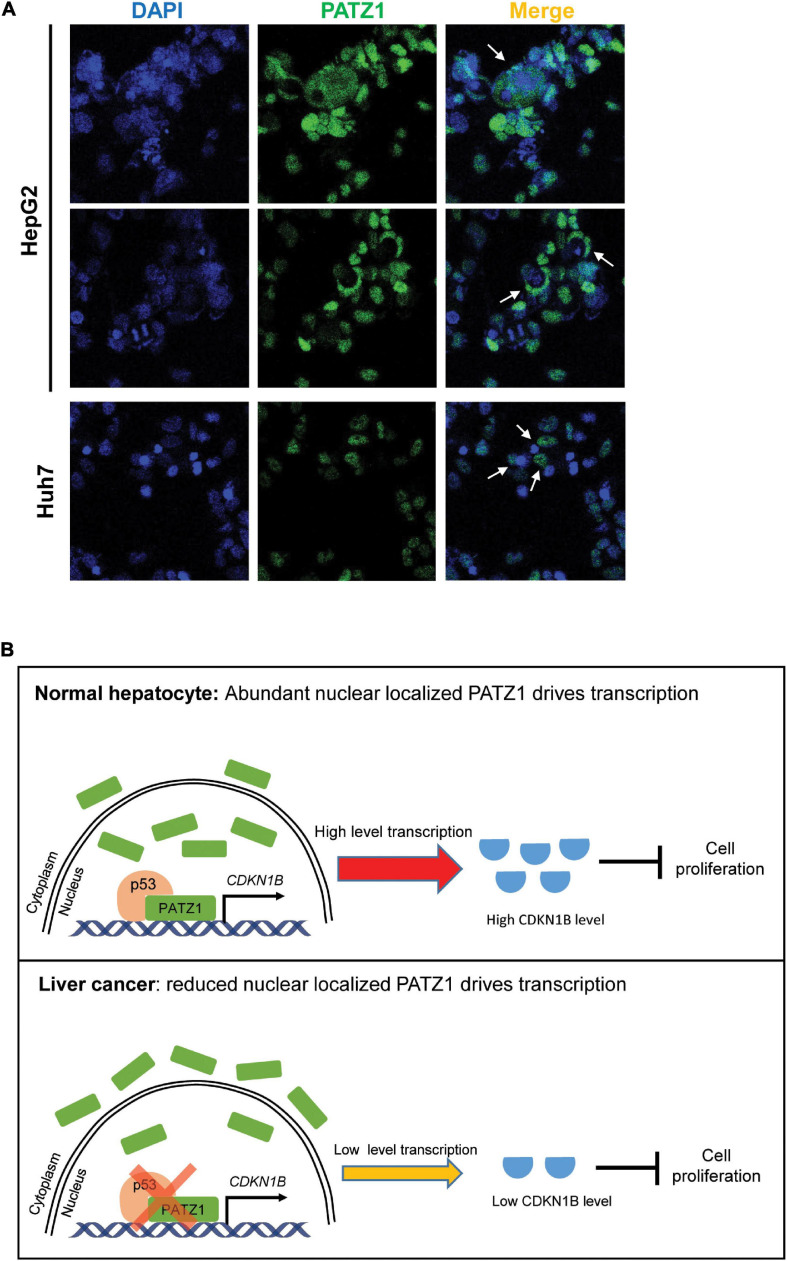
Schematic illustration of PATZ1 in normal hepatocytes and liver cancer. **(A)** Cytoplasmic localization of PATZ1 was observed in HepG2 and Huh7 cells as indicated by the white arrows. PATZ1 protein levels were quantified by immunofluorescence and DAPI was used as a nuclear control. **(B)** Top: In normal hepatocytes, PATZ1 is localized in the nucleus and binds to the promoter of *CDKN1B* to promote its transcriptional activation, resulting in a high expression level of CDKN1B which inhibits cell proliferation by suppressing CDK2-Cyclin E. Bottom: In liver cancer cells, PATZ1 is aberrantly delocalized to the cytoplasm and accordingly the level of nuclear PATZ1 is reduced. This restrains PATZ1-mediated transcriptional activation of CDKN1B and reduces the CDKN1B-mediated inhibition of cell proliferation.

In summary, we have demonstrated that PATZ1 inhibits cell proliferation by promoting CDKN1B expression, thereby indicating that PATZ1 functions as a tumor suppressor in liver cancer. Remarkably, PATZ1 is delocalized to the cytoplasm in the HCC cell lines (Figure 6A). Thus, the cytoplasmic delocalization of PATZ1 tumor suppressor in the HCC cell lines might negate its nuclear transcriptional activation of CDKN1B, thereby resulting in reduced expression of CDKN1B. Subsequently, this may potentiate oncogenesis through augmented cell proliferation. A summary of the role of PATZ1 in liver cancer is illustrated in Figure 6B.

## Discussion

To date, the role of PATZ1 in cancer progression has been studied in breast, lung, thyroid, testicular cancers and lymphomas (Mastrangelo et al., 2000; Pan et al., 2011; Valentino et al., 2013; Guadagno et al., 2017). However, there remains a paucity of data on the involvement of PATZ1 in liver cancer. Here, we investigated the role of PATZ1 in liver cancer, which has poor mechanistic insights despite well-defined etiologies (Møller, 2000). We found a high expression of PATZ1 in HCC cell lines while PATZ1 was weakly expressed in normal hepatocytes (Figure 1). Thus, we aimed to investigate the role of PATZ1 in HCC.

In this study, we employed holistic genome-wide approaches to systematically examine the role of PATZ1 in HCC. Our PATZ1 ChIP-seq data revealed that PATZ1 binds to the genomic loci of many cancer-related genes in HepG2 cells (Supplementary Figure 4). In addition, functional annotation of PATZ1 binding sites and related genes revealed a strong enrichment in GO terms associated with cell cycle and its regulation (Figures 1F, 3D). Similarly, RNA-seq analysis by in *Patz1*^–/–^ mouse embryonic fibroblasts (MEFs) found enrichment for genes associated with cell proliferation (Keskin et al., 2015). Overall these data strongly support the notion that PATZ1 plays a crucial role in cancer through regulation of cell cycle and proliferation genes. Indeed, we found that the depletion of PATZ1 in HepG2 cells led to increase in cell viability, colony formation ability and elevated S-phase entry (Figure 2).

Importantly, we have identified CDKN1B as a key mediator of PATZ1-modulated cell proliferation. CDKN1B is a cyclin-dependent kinase (CDK) inhibitor that belongs to the Cip/Kip family of CDK inhibitors (CKIs). CDKN1B functions to suppress G_1_-S progression of the cell cycle by inhibiting Cyclin E-CDK2 (Tannapfel et al., 2000). Clinically, low expression of CDKN1B has been associated with advanced stage of hepatocellular carcinoma while elevated CDKN1B expression serves as a favorable prognostic marker for better HCC survival rate (Ito et al., 1999; Matsuda et al., 2013; Niu et al., 2015; Xu et al., 2015). Reduced CDKN1B expression has been found to be a significant prognostic factor in other cancers (Chu et al., 2008). Given the dysregulation of CDKN1B in various cancers, the knowledge of the direct regulation of CDKN1B by PATZ1 provides a promising perspective in tackling tumor development and progression.

Interestingly, the direct regulation of CDKN1B by PATZ1 was abrogated in the absence of p53. The interaction between PATZ1 and p53 indicates a possible role of p53 in modulating the molecular role of PATZ1. In the p53-null Hep3B cell, PATZ1 knockdown resulted in a reduced transcript level but not the protein level of CDKN1B. Similarly, PATZ1 was found to be able to regulate the transcription of p53-dependent genes in the absence of p53 in p53-null Saos-2 and H1299 cells (Valentino et al., 2013). The unaltered CDKN1B protein expression in PATZ1-interfered Hep3B cells suggests a post-transcriptional regulation of CDKN1B which is dependent on p53 status. p53 has been previously shown to modulate the ability of PATZ1 to regulate CDKN1A expression, which is also a member of the Cip/Kip family of CKIs (Valentino et al., 2013). Notably, PATZ1 was found to bind to the promoter region of CDKN1B independent of p53 in Hep3B cells, suggesting a role of p53 in regulating the function of PATZ1. Furthermore, our study has found that PATZ1 and p53 can interact and tend to be highly enriched at the TSS, therefore indicating a strong association between PATZ1 and p53. Similarly, increased cell population in the S phase was previously reported in p53 siRNA-treated HepG2 cells, which is similar with the PATZ1-depleted HepG2 cells in our studies, thereby indicating analogous phenotype between PATZ1 and p53 depletion. The mechanistic and functional synergy between PATZ1 and p53 offers a new perspective into the role of p53 in cancer. Further studies can elucidate novel targets of the PATZ1-p53 transcriptional complex.

The cellular delocalization of PATZ1 parallels the enhanced cytoplasmic localization of PATZ1 with increasing malignancy in testicular tumor, where PATZ1 was found to be a tumor suppressor (Fedele et al., 2008). Interestingly, cytoplasmic localization of tumor suppressor proteins was previously found to promote carcinogenesis in human cancer (Viglietto et al., 2002). This might possibly account for the high expression of PATZ1 in HCC cells, of which cytoplasmic localization might drive further progression of liver cancer. It was previously suggested that aberrant cytoplasmic delocalization of PATZ1 might disrupt its nuclear transcriptional role or lead to the acquisition of a novel cytoplasmic role (Fedele et al., 2008). Further investigations would be required to address the cytoplasmic role of PATZ1 in liver cancer progression.

Notably, by mapping these differentially-expressed genes with the PATZ1 putative target genes, we found that PATZ1 depletion results in a substantially greater number of putative targets that were upregulated (Figure 3E). We thus reckon that PATZ1 primarily functions as a transcriptional repressor in HepG2 cells which might be attributed to its POZ domain since it has been shown that the POZ domain is crucial for transcriptional repression (Collins et al., 2001). Further, the POZ domain has been found to interact with the co-repressors N-CoR, SMRT and HDAC complexes (Huynh and Bardwell, 1998; Guenther et al., 2001). Interestingly, our ChIP-seq data indicates that PATZ1 binds to promoter regions of various HDAC genes (Supplementary Table 4). Together, these indicate a possible involvement of PATZ1 in epigenetic suppression of gene expression.

## Conclusion

We have demonstrated that PATZ1 cooperates with p53 and regulates liver cancer cell proliferation by directly regulating *CDKN1B*. With the strongly established role of CDKN1B as a prognostic marker in various cancer types, our discovery of PATZ1 as a potent regulator might open new possibilities for better management of tumor progression.

## Data Availability Statement

The datasets generated for this study can be found in online repositories. The names of the repository/repositories and accession number(s) can be found in the article/Supplementary Material.

## Author Contributions

QW conceived and designed the experiments. ZN, JL, JS, GJ, MH, XL, and CP performed the experiments and analyzed the data. SY, YC, YoZ, SW, HY, YiZ, YL, and ZJ contributed reagents, materials, and analysis tools. ZN and QW wrote the manuscript. All authors read and approved the final manuscript.

## Conflict of Interest

The authors declare that the research was conducted in the absence of any commercial or financial relationships that could be construed as a potential conflict of interest. The reviewer Y-CL declared a shared affiliation with several of the authors to the handling editor at the time of review.

## Footnotes

1 http://meme.nbcr.net/meme/cgi-bin/meme-chip.cgi

## Supplementary Material

Supplementary Figure 1 Validation of gene expression microarray. **(A)** qRT-PCR was performed on arbitrarily selected differentially expressed genes and normalized to mRNA level of β-actin. Fold changes were computed in terms of percentage fold change of the siPATZ1-treated transcriptome relative to the siCTRL-treated transcriptome. Data was presented as mean ± SEM; *n* = 2. **(B)** Thumbnail-dendrogram representing the corresponding downregulated gene targets in siCTRL- and siPATZ1-treated HepG2 cells. **(C)** Thumbnail-dendrogram representing the corresponding upregulated gene targets in siCTRL- and siPATZ1-treated HepG2 cells. The color scale bar below presents the fold change.

Supplementary Figure 2 GO clustering of the transcriptome of siCTRL- and siPATZ1-treated HepG2 cells. Gene expression microarray analysis of siCTRL- and siPATZ1-treated HepG2 cell duplicates were subjected to log2-transformed gene expression followed by *K*-means clustering. Fold-change threshold of 1.4 is applied. Data is represented in a thumbnail-dendrogram format. **(A)** GO clustering of differentially-expressed genes in the Cip/Kip family. The color scale bar below presents the fold change. **(B)** KEGG pathway analysis of differentially expressed genes (*P* ≤ 0.05). Numerical values on the right indicate the number of genes associated with the respective KEGG pathway.

Supplementary Figure 3 Validation of PATZ1 ChIP-Seq targets. qRT-PCR was performed with input DNA and PATZ1 ChIP-DNA on arbitrarily selected PATZ1 ChIP-Seq targets and normalized to the expression of p2. The ChIP-qPCR enrichment fold changes are indicated at the top of the respective bars while the corresponding ChIP-Seq fold changes are indicated at the bottom. Data was presented as mean ± SEM; *n* = 2.

Supplementary Figure 4 Overlap of PATZ1 putative ChIP-Seq targets with oncogenic signatures. Venn diagram indicating the overlap between PATZ1 putative targets and the C6 oncogenic signature from the Molecular Signatures Database. 1,624 (54%) of the putative PATZ1 targets are cancer-associated genes.

Supplementary Figure 5 Conserved binding of PATZ1 to CDKN1B promoter region. ChIP-qPCR results validate the binding of PATZ1 to the promoter region of CDKN1B. **(A)** Locations of real-time PCR primers (amplicons 1 to 8) were mapped to the promoter regions of CDKN1B promoter. ChIP was performed with antibody against PATZ1 and the resulting ChIP-DNA was analyzed by qRT-PCR with the primers against amplicons 1 to 8 of CDKN1B. High enrichment of PATZ1 to amplicon 5 of CDKN1B in HCT116 cells **(B)**, h1 hESCs **(C)** and Hep3B cells **(D)** were found, which correspond to the region TSS +9/+168 of the CDKN1B gene. Enrichment fold were computed from the apparent IP efficiency (ratio of ChIP-DNA over input DNA). Data was presented as mean ± SEM; *n* = 2. **(E)** DNA sequence of CDKN1B promoter region (−93 to +297). Nucleotides in blue: putative p53 binding sites; Nucleotides in green: PATZ1 binding site; T in red: transcriptional start site. **(F)** Enhanced enrichment of sequential ChIP (PATZ1 and p53) DNA at CDKN1B promoter region.

Supplementary Figure 6 Co-immunostaining of PATZ1 and p53 in HCC cell lines. PATZ1 was observed in HepG2 and Hep3B cells while p53 was not expressed in Hep3B cells.

Supplementary Table 1 Primers used for qRT-PCR.

Supplementary Table 2 Primers used for ChIP-qPCR.

Supplementary Table 3 Top 60 PATZ1 ChIP-seq targets.

Supplementary Table 4 List of HDAC isoforms enriched in PATZ1 ChIP-Seq analysis.

## References

[B1] Abramova A. Sakaguchi S. Schebesta A. Hassan H. Boucheron N. Valent P. (2013). The transcription factor MAZR preferentially acts as a transcriptional repressor in mast cells and plays a minor role in the regulation of effector functions in response to FcepsilonRI stimulation. *PLoS One.* 8:e77677. 10.1371/journal.pone.0077677 24204913 PMC3804165

[B2] Ahn E. Y. Kim J. S. Kim G. J. Park Y. N. (2013). RASSF1A-mediated regulation of AREG via the Hippo pathway in hepatocellular carcinoma. *Mol. Cancer Res.* 11 748–758. 10.1158/1541-7786.MCR-12-0665 23594797

[B3] Ananthakrishnan A. Gogineni V. Saeian K. (2006). Epidemiology of Primary and Secondary Liver Cancers. *Semin Interv. Rad.* 23 47–63. 10.1055/s-2006-939841 21326720 PMC3036307

[B4] Bai X. L. Zhang Q. Ye L. Y. Liang F. Sun X. Chen Y. (2015). Myocyte enhancer factor 2C regulation of hepatocellular carcinoma via vascular endothelial growth factor and Wnt/beta-catenin signaling. *Oncogene* 34 4089–4097. 10.1038/onc.2014.337 25328135

[B5] Barrett T. Wilhite S. E. Ledoux P. Evangelista C. Kim I. F. Tomashevsky M. (2013). NCBI GEO: archive for functional genomics data sets–update. *Nucleic Acids Res.* 41 D991–D995. 10.1093/nar/gks1193 23193258 PMC3531084

[B6] Bilic I. Koesters C. Unger B. Sekimata M. Hertweck A. Maschek R. (2006). Negative regulation of CD8 expression via Cd8 enhancer-mediated recruitment of the zinc finger protein MAZR. *Nat. Immunol.* 7 392–400. 10.1038/ni1311 16491076 PMC3001192

[B7] Chacko S. Samanta S. (2016). Hepatocellular carcinoma: A life-threatening disease. *Biomed. Pharmacother.* 84 1679–1688. 10.1016/j.biopha.2016.10.078 27823920

[B8] Chiappetta G. Valentino T. Vitiello M. Pasquinelli R. Monaco M. Palma G. (2015). PATZ1 acts as a tumor suppressor in thyroid cancer via targeting p53-dependent genes involved in EMT and cell migration. *Oncotarget* 6 5310–5323. 10.18632/oncotarget.2776 25595894 PMC4467151

[B9] Cho J. H. Kim M. J. Kim K. J. Kim J. R. (2012). POZ/BTB and AT-hook-containing zinc finger protein 1 (PATZ1) inhibits endothelial cell senescence through a p53 dependent pathway. *Cell Death Diff.* 19 703–712. 10.1038/cdd.2011.142 22052190 PMC3307983

[B10] Chu I. M. Hengst L. Slingerland J. M. (2008). The Cdk inhibitor p27 in human cancer: prognostic potential and relevance to anticancer therapy. *Nat. Rev. Cancer* 8 253–267. 10.1038/nrc2347 18354415

[B11] Collins T. R. Stone J. R. Williams A. J. (2001). All in the Family: the BTB/POZ, KRAB, and SCAN Domains. *Mol. Cell Biol.* 21 3609–3615. 10.1128/MCB.21.11.3609-3615.2001 11340155 PMC86980

[B12] Eisen M. B. Spellman P. T. Brown P. O. Botstein D. (1998). Cluster analysis and display of genome-wide expression patterns. *Proc. Natl. Acad. Sci. U. S. A.* 95 14863–14868. 10.1073/pnas.95.25.14863 9843981 PMC24541

[B13] El-Serag H. B. (2011). Hepatocellular carcinoma. *N. Engl. J. Med.* 365 1118–1127. 10.1056/NEJMra1001683 21992124

[B14] El-Serag H. B. (2012). Epidemiology of viral hepatitis and hepatocellular carcinoma. *Gastroenterology* 142 1264–1273. 10.1053/j.gastro.2011.12.061 22537432 PMC3338949

[B15] Esposito F. Boscia F. Franco R. Fusco A. Kitazawa S. Looijenga L. H. (2011). Down-regulation of oestrogen receptor-beta associates with transcriptional co-regulator PATZ1 delocalization in human testicular seminomas. *J. Pathol.* 224 110–120. 10.1002/path.2846 21381029

[B16] Fedele M. Franco R. Salvatore G. Paronetto M. P. Barbagallo F. Pero R. (2008). PATZ1 gene has a critical role in the spermatogenesis and testicular tumours. *J. Pathol.* 215 39–47. 10.1002/path.2323 18241078

[B17] Flores A. Marrero J. A. (2014). Emerging Trends in Hepatocellular Carcinoma: Focus on Diagnosis and Therapeutics. *Clin. Med. Insights Oncol*. 8 71–76. 10.4137/CMO.S9926 24899827 PMC4039215

[B18] Franco R. Scognamiglio G. Valentino E. Vitiello M. Luciano A. Palma G. (2016). PATZ1 expression correlates positively with BAX and negatively with BCL6 and survival in human diffuse large B cell lymphomas. *Oncotarget* 7 59158–59172. 10.18632/oncotarget.10993 27494852 PMC5312302

[B19] Guadagno E. Vitiello M. Francesca P. Calì G. Caponnetto F. Cesselli D. (2017). PATZ1 is a new prognostic marker of glioblastoma associated with the stem-like phenotype and enriched in the proneural subtype. *Oncotarget* 8 59282–59300. 10.18632/oncotarget 28938636 PMC5601732

[B20] Guenther M. G. Barak O. Lazar M. A. (2001). The SMRT and N-CoR corepressors are activating cofactors for histone deacetylase 3. *Mol. Cell Biol.* 21 6091–6101. 10.1128/MCB.21.18.6091-6101.2001 11509652 PMC87326

[B21] Heinz S. Benner C. Spann N. Bertolino E. Lin Y. C. Laslo P. (2010). Simple combinations of lineage-determining transcription factors prime cis-regulatory elements required for macrophage and B cell identities. *Mol. Cell.* 38 576–589. 10.1016/j.molcel.2010.05.004 20513432 PMC2898526

[B22] Ho M. Y. Liang C. M. Liang S. M. (2016). PATZ1 induces PP4R2 to form a negative feedback loop on IKK/NF-kappaB signaling in lung cancer. *Oncotarget* 7 52255–52269. 10.18632/oncotarget.10427 27391343 PMC5239549

[B23] Huang D. W. Sherman B. T. Lempicki R. A. (2009a). Bioinformatics enrichment tools: paths toward the comprehensive functional analysis of large gene lists. *Nucleic Acids Res.* 37 1–13. 10.1093/nar/gkn923 19033363 PMC2615629

[B24] Huang D. W. Sherman B. T. Lempicki R. A. (2009b). Systematic and integrative analysis of large gene lists using DAVID bioinformatics resources. *Nat. Protoc.* 4 44–57. 10.1038/nprot.2008.211 19131956

[B25] Huynh K. D. Bardwell V. J. (1998). The BCL-6 POZ domain and other POZ domains interact with the co-repressors N-CoR and SMRT. *Oncogene* 17 2473–2484. 10.1038/sj.onc.1202197 9824158

[B26] Inayat F. Rahman U. Z. Hurairah A. (2016). Hepatocellular carcinoma in nonalcoholic fatty liver disease. *Cureus* 8:e754. 10.7759/cureus.754 27733959 PMC5045331

[B27] Ito Y. Matsuura N. Sakon M. Miyoshi E. Noda K. Takeda T. (1999). Expression and prognostic roles of the G1-S modulators in hepatocellular carcinoma: p27 independently predicts the recurrence. *Hepatology* 30 90–99. 10.1002/hep.510300114 10385644

[B28] Jamieson A. C. Wang H. Kim S. H. (1996). A zinc finger directory for high-affinity DNA recognition. *Proc. Natl. Acad. Sci. U. S. A.* 93 12834–12839. 10.1073/pnas.93.23.12834 8917505 PMC24006

[B29] Janku F. Kaseb A. O. Tsimberidou A. M. Wolff R. A. Kurzrock R. (2014). Identification of novel therapeutic targets in the PI3K/AKT/mTOR pathway in hepatocellular carcinoma using targeted next generation sequencing. *Oncotarget* 5 3012–3022. 10.18632/oncotarget.1687 24931142 PMC4102787

[B30] Kelley R. K. Venook A. P. (2013). Novel therapeutics in hepatocellular carcinoma: how can we make progress? *Am. Soc. Clin. Oncol. Educ. Book* 33:e137 10.1200/EdBook_AM.2013.33.e137 23714481

[B31] Keskin N. Deniz E. Eryilmaz J. Un M. Batur T. Ersahin T. (2015). PATZ1 Is a DNA Damage-Responsive Transcription Factor That Inhibits p53 Function. *Mol. Cell Biol.* 35 1741–1753. 10.1128/MCB.01475-14 25755280 PMC4405645

[B32] Kobayashi A. Yamagiwa H. Hoshino H. Muto A. Sato K. Morita M. (2000). A combinatorial code for gene expression generated by transcription factor Bach2 and MAZR (MAZ-related factor) through the BTB/POZ domain. *Mol. Cell Biol.* 20 1733–1746. 10.1128/mcb.20.5.1733-1746.2000 10669750 PMC85356

[B33] Lanaya H. Natarajan A. Komposch K. Li L. Amberg N. Chen L. (2014). EGFR has a tumour-promoting role in liver macrophages during hepatocellular carcinoma formation. *Nat. Cell Biol.* 16 972–977. 10.1038/ncb3031 25173978 PMC4183558

[B34] Lee S. U. Maeda T. (2012). POK/ZBTB proteins: an emerging family of proteins that regulate lymphoid development and function. *Immunol. Rev.* 247 107–119. 10.1111/j.1600-065X.2012.01116.x 22500835 PMC3334328

[B35] Lee Y. H. Ma H. Tan T. Z. Swee Siang, Ng S. S. Soong R. (2012). Protein arginine methyltransferase 6 regulates embryonic stem cell identity. *Stem Cells Dev.* 21 2613–2622. 10.1089/scd.2011.0330 22455726 PMC5729635

[B36] Li H. (2014). Toward better understanding of artifacts in variant calling from high-coverage samples. *Bioinformatics* 30 2843–2851. 10.1093/bioinformatics/btu356 24974202 PMC4271055

[B37] Ma H. Ng H. M. Teh X. Li H. Lee Y. H. Chong Y. M. (2014a). Zfp322a regulates mouse ES cell pluripotency and enhances reprogramming efficiency. *PLoS Genet.* 10:e1004038. 10.1371/journal.pgen.1004038 24550733 PMC3923668

[B38] Ma H. Ow J. R. Tan B. C. Goh Z. Feng B. Loh Y. H. (2014b). The dosage of Patz1 modulates reprogramming process. *Sci. Rep.* 4:7519. 10.1038/srep07519 25515777 PMC4268633

[B39] Mastrangelo T. Modena P. Tornielli S. Bullrich F. Testi M. A. Mezzelani A. (2000). A novel zinc finger gene is fused to EWS in small round cell tumor. *Oncogene* 19 3799–3804. 10.1038/sj.onc.1203762 10949935

[B40] Matsuda Y. Wakai T. Hirose Y. Osawa M. Fujimaki S. Kubota M. (2013). p27 is a critical prognostic biomarker in non-alcoholic steatohepatitis-related hepatocellular Carcinoma. *Int. J. Mol. Sci.* 14 23499–23515. 10.3390/ijms141223499 24351862 PMC3876059

[B41] Møller M. B. (2000). p27 in cell cycle control and cancer. *Leuk. Lymphoma.* 39 19–27. 10.3109/10428190009053535 10975380

[B42] Niu Z. S. Niu X. J. Wang M. (2015). Management of hepatocellular carcinoma: Predictive value of immunohistochemical markers for postoperative survival. *World J. Hepatol.* 7 7–27. 10.4254/wjh.v7.i1.7 25624992 PMC4295195

[B43] Ow J. R. Ma H. Jean A. Lee Y. H. Chong Y. M. Soong R. (2014). Patz1 regulates embryonic stem cell identity. *Stem Cells Dev.* 23 1062–1073. 10.1089/scd.2013.0430 24380431

[B44] Pan H. Fu X. Huang W. (2011). Molecular mechanism of liver cancer. *Anti Cancer Agents Med. Chem.* 11 493–499. 10.2174/187152011796011073 21554201

[B45] Parkin D. M. Bray F. Ferlay J. Pisani P. (2005). Global cancer statistics, 2002. *CA Cancer J. Clin.* 55 74–108. 10.3322/canjclin.55.2.74 15761078

[B46] Sakaguchi S. Hainberger D. Tizian C. Tanaka H. Okuda T. Taniuchi I. (2015). MAZR and Runx factors synergistically repress ThPOK during CD8+ T cell lineage development. *J. Immunol.* 195 2879–2887. 10.4049/jimmunol.1500387 26254341

[B47] Sakaguchi S. Hombauer M. Bilic I. Naoe Y. Schebesta A. Taniuchi I. (2010). The zinc-finger protein MAZR is part of the transcription factor network that controls the CD4 versus CD8 lineage fate of double-positive thymocytes. *Nat. Immunol.* 11 442–448. 10.1038/ni.1860 20383150 PMC3365445

[B48] Saldanha A. J. (2004). Java Treeview—extensible visualization of microarray data. *Bioinformatics* 20 3246–3248. 10.1093/bioinformatics/bth349 15180930

[B49] Shen L. Shao N. Liu X. Nestler E. (2014). ngs.plot: Quick mining and visualization of next-generation sequencing data by integrating genomic databases. *BMC Genom.* 15:284. 10.1186/1471-2164-15-284 24735413 PMC4028082

[B50] Sherr C. J. Roberts J. M. (1999). CDK inhibitors: positive and negative regulators of G1-phase progression. *Genes Dev.* 13 1501–1512. 10.1101/gad.13.12.1501 10385618

[B51] Tannapfel A. Grund D. Katalinic, Uhlmann A. D. Köckerling F. Haugwitz U. (2000). Decreased expression of p27 protein is associated with advanced tumor stage in hepatocellular carcinoma. *Int. J. Cancer* 89 350–355. 10.1002/1097-0215(20000720)89:4 10956409

[B52] Tian X. Sun D. Zhang Y. Zhao S. Xiong H. Fang J. (2008). Zinc finger protein 278, a potential oncogene in human colorectal cancer. *Acta Biochim. Biophys. Sin.* 40 289–296. 10.1111/j.1745-7270.2008.00405.x 18401526

[B53] Uhlen M. Fagerberg L. Hallstrom B. M. Lindskog C. Oksvold P. Mardinoglu A. (2015). Proteomics tissue-based map of the human proteome. *Science* 347:1260419. 10.1126/science.1260419 25613900

[B54] Valentino T. Palmieri D. Vitiello M. Pierantoni G. M. Fusco A. Fedele M. (2013). PATZ1 interacts with p53 and regulates expression of p53-target genes enhancing apoptosis or cell survival based on the cellular context. *Cell Death Dis.* 4:e963. 10.1038/cddis.2013.500 24336083 PMC3877567

[B55] Viglietto G. Motti M. L. Fusco A. (2002). Understanding p27(kip1) deregulation in cancer: down-regulation or mislocalization. *Cell Cycle* 1 394–400. 10.4161/cc.1.6.263 12548012

[B56] Vitiello M. Valentino T. De Menna M. Crescenzi E. Francesca P. Rea D. (2016). PATZ1 is a target of miR-29b that is induced by Ha-Ras oncogene in rat thyroid cells. *Sci. Rep.* 6:25268. 10.1038/srep25268 27125250 PMC4850481

[B57] Wang D. Han S. Peng R. Jiao C. Wang X. Han Z. (2014). DUSP28 contributes to human hepatocellular carcinoma via regulation of the p38 MAPK signaling. *Int. J. Oncol.* 45 2596–2604. 10.3892/ijo.2014.2653 25230705

[B58] Xu F. Yang J. Chen J. Wu Q. Gong W. Zhang J. (2015). Differential co-expression and regulation analyses reveal different mechanisms underlying major depressive disorder and subsyndromal symptomatic depression. *BMC Bioinform.* 16:112. 10.1186/s12859-015-0543-y 25880836 PMC4434877

[B59] Zhang Y. Liu T. Meyer C. A. Eeckhoute J. Johnson D. S. Bernsteinet B. E. (2008). Model-based analysis of ChIP-Seq (MACS). *Genome Biol.* 9:R137. 10.1186/gb-2008-9-9-r137 18798982 PMC2592715

