# Supplementary figures and images for: PATZ1 (MAZR) Co-occupies Genomic Sites With p53 and Inhibits Liver Cancer Cell Proliferation via Regulating p27
Source: Front Cell Dev Biol. 2021 Feb 1;9:586150. doi: 10.3389/fcell.2021.586150 (PMC7882738; doi:10.3389/fcell.2021.586150)

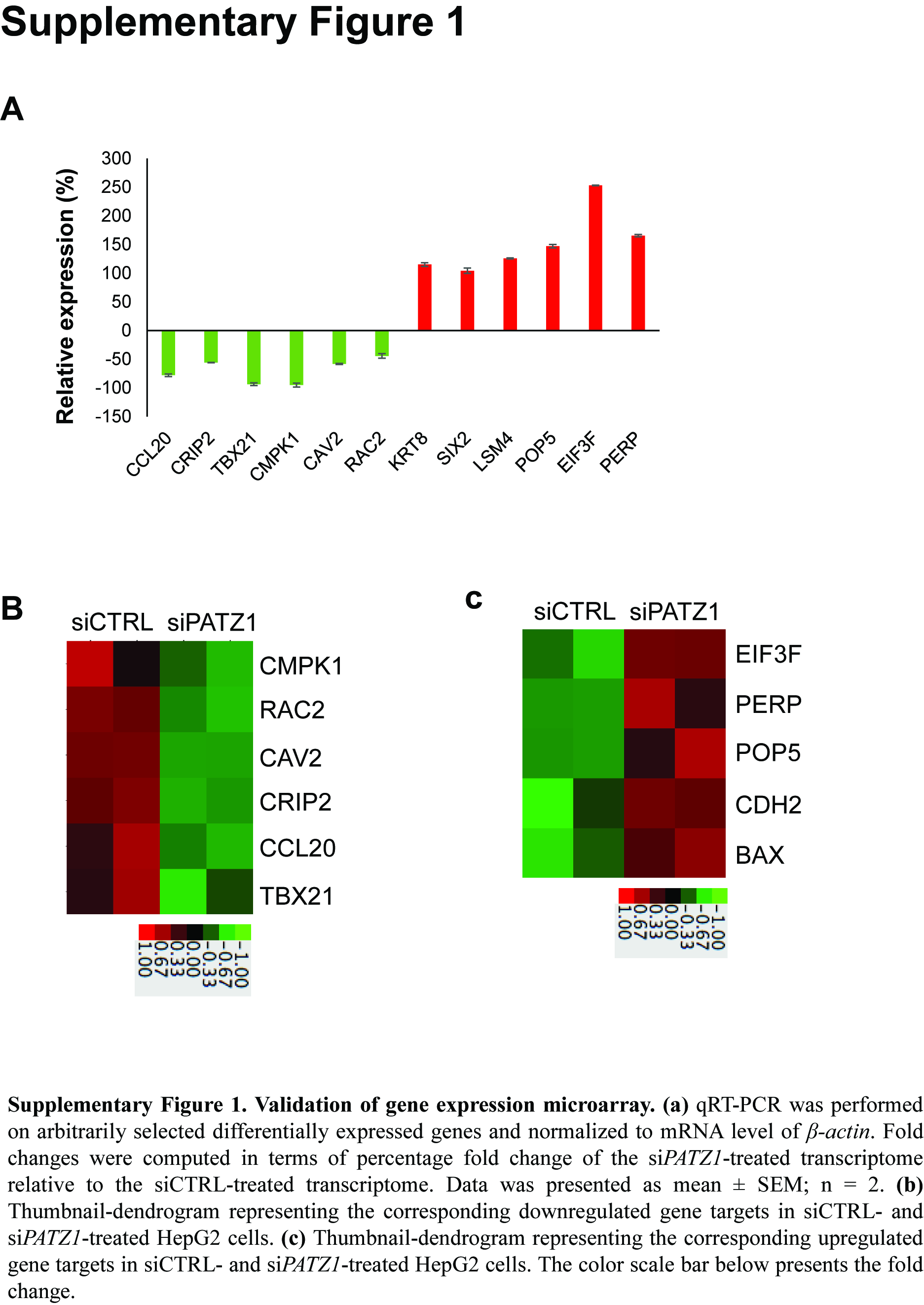

Supplement: Supplementary Figure 1 — Validation of gene expression microarray. (A) qRT-PCR was performed on arbitrarily selected differentially expressed genes and normalized to mRNA level of β-actin. Fold changes were computed in terms of percentage fold change of the siPATZ1-treated transcriptome relative to the siCTRL-treated transcriptome. Data was presented as mean ± SEM; n = 2. (B) Thumbnail-dendrogram representing the corresponding downregulated gene targets in siCTRL- and siPATZ1-treated HepG2 cells. (C) Thumbnail-dendrogram representing the corresponding upregulated gene targets in siCTRL- and siPATZ1-treated HepG2 cells. The color scale bar below presents the fold change. [file Image_1.TIF]

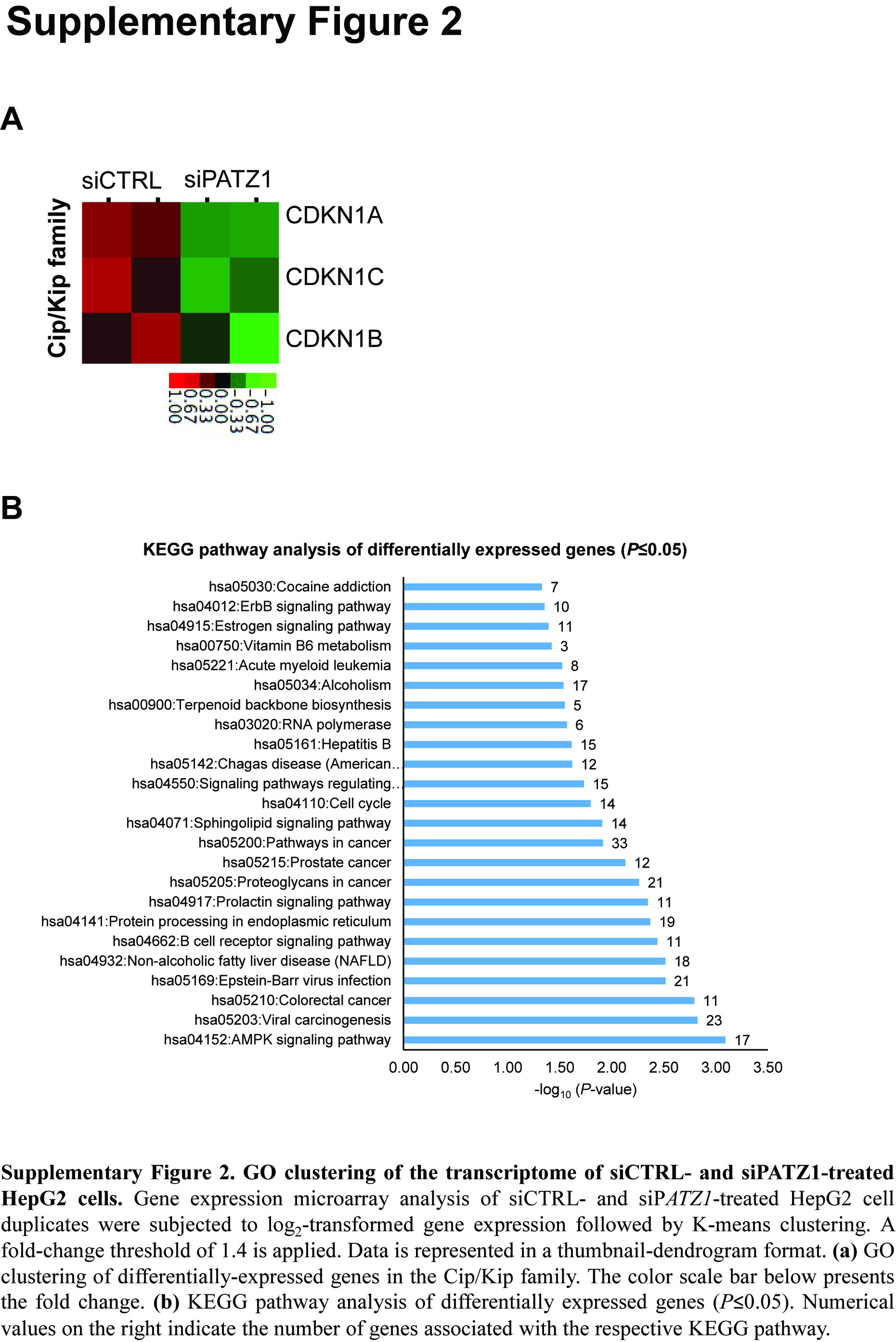

Supplement: Supplementary Figure 2 — GO clustering of the transcriptome of siCTRL- and siPATZ1-treated HepG2 cells. Gene expression microarray analysis of siCTRL- and siPATZ1-treated HepG2 cell duplicates were subjected to log2-transformed gene expression followed by K-means clustering. Fold-change threshold of 1.4 is applied. Data is represented in a thumbnail-dendrogram format. (A) GO clustering of differentially-expressed genes in the Cip/Kip family. The color scale bar below presents the fold change. (B) KEGG pathway analysis of differentially expressed genes (P ≤ 0.05). Numerical values on the right indicate the number of genes associated with the respective KEGG pathway. [file Image_2.TIF]

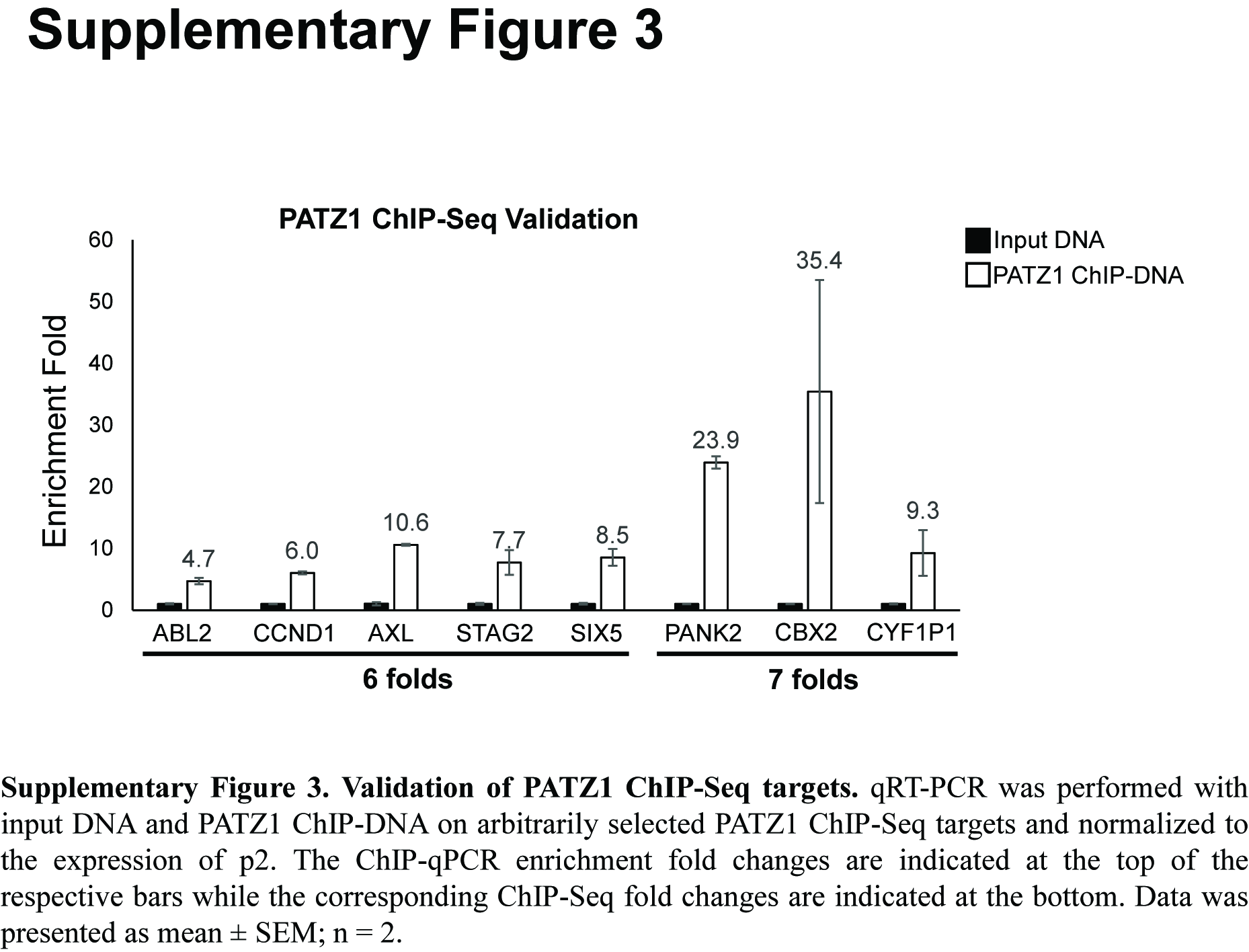

Supplement: Supplementary Figure 3 — Validation of PATZ1 ChIP-Seq targets. qRT-PCR was performed with input DNA and PATZ1 ChIP-DNA on arbitrarily selected PATZ1 ChIP-Seq targets and normalized to the expression of p2. The ChIP-qPCR enrichment fold changes are indicated at the top of the respective bars while the corresponding ChIP-Seq fold changes are indicated at the bottom. Data was presented as mean ± SEM; n = 2. [file Image_3.TIF]

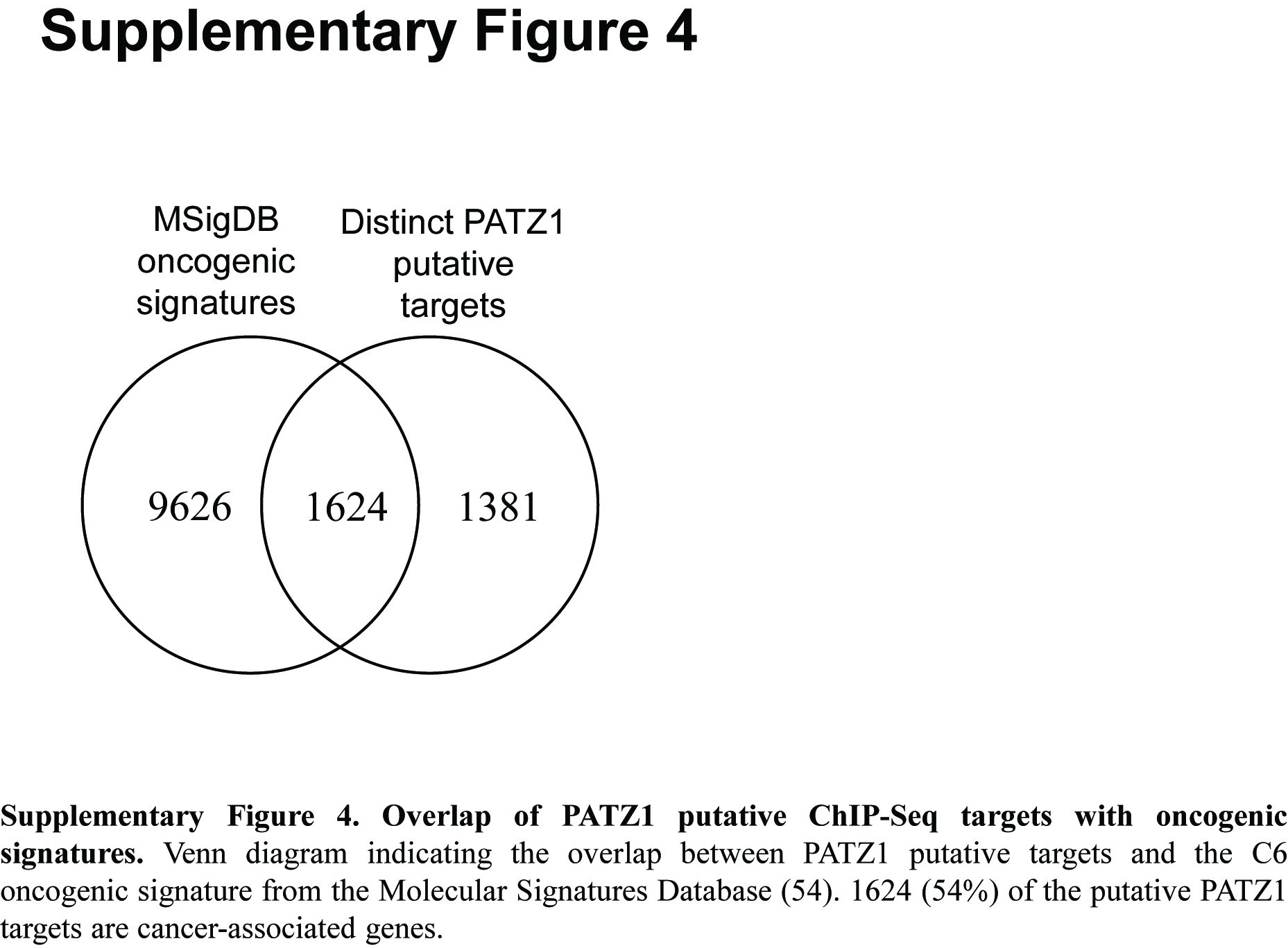

Supplement: Supplementary Figure 4 — Overlap of PATZ1 putative ChIP-Seq targets with oncogenic signatures. Venn diagram indicating the overlap between PATZ1 putative targets and the C6 oncogenic signature from the Molecular Signatures Database. 1,624 (54%) of the putative PATZ1 targets are cancer-associated genes. [file Image_4.TIF]

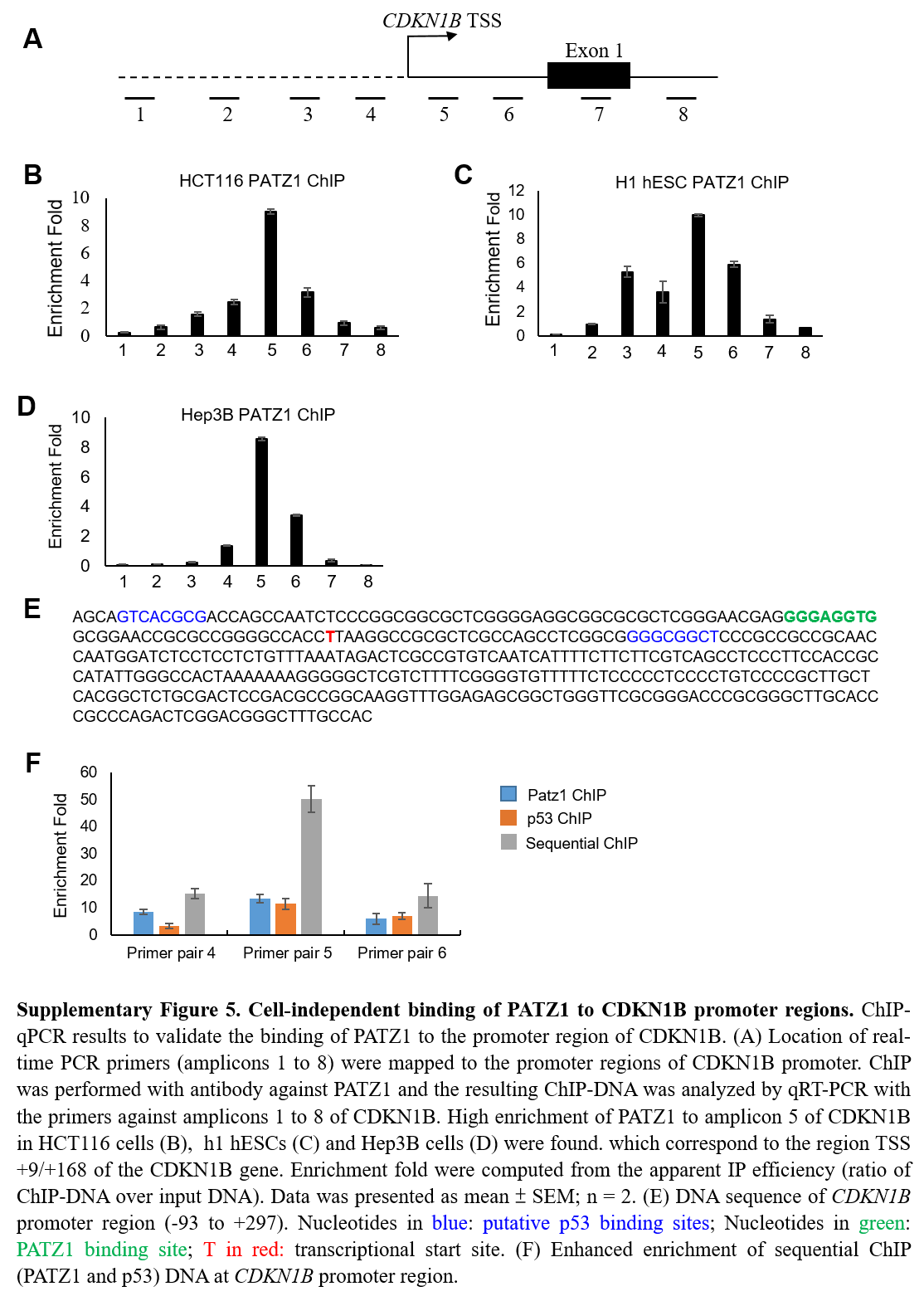

Supplement: Supplementary Figure 5 — Conserved binding of PATZ1 to CDKN1B promoter region. ChIP-qPCR results validate the binding of PATZ1 to the promoter region of CDKN1B. (A) Locations of real-time PCR primers (amplicons 1 to 8) were mapped to the promoter regions of CDKN1B promoter. ChIP was performed with antibody against PATZ1 and the resulting ChIP-DNA was analyzed by qRT-PCR with the primers against amplicons 1 to 8 of CDKN1B. High enrichment of PATZ1 to amplicon 5 of CDKN1B in HCT116 cells (B), h1 hESCs (C) and Hep3B cells (D) were found, which correspond to the region TSS +9/+168 of the CDKN1B gene. Enrichment fold were computed from the apparent IP efficiency (ratio of ChIP-DNA over input DNA). Data was presented as mean ± SEM; n = 2. (E) DNA sequence of CDKN1B promoter region (−93 to +297). Nucleotides in blue: putative p53 binding sites; Nucleotides in green: PATZ1 binding site; T in red: transcriptional start site. (F) Enhanced enrichment of sequential ChIP (PATZ1 and p53) DNA at CDKN1B promoter region. [file Image_5.TIF]

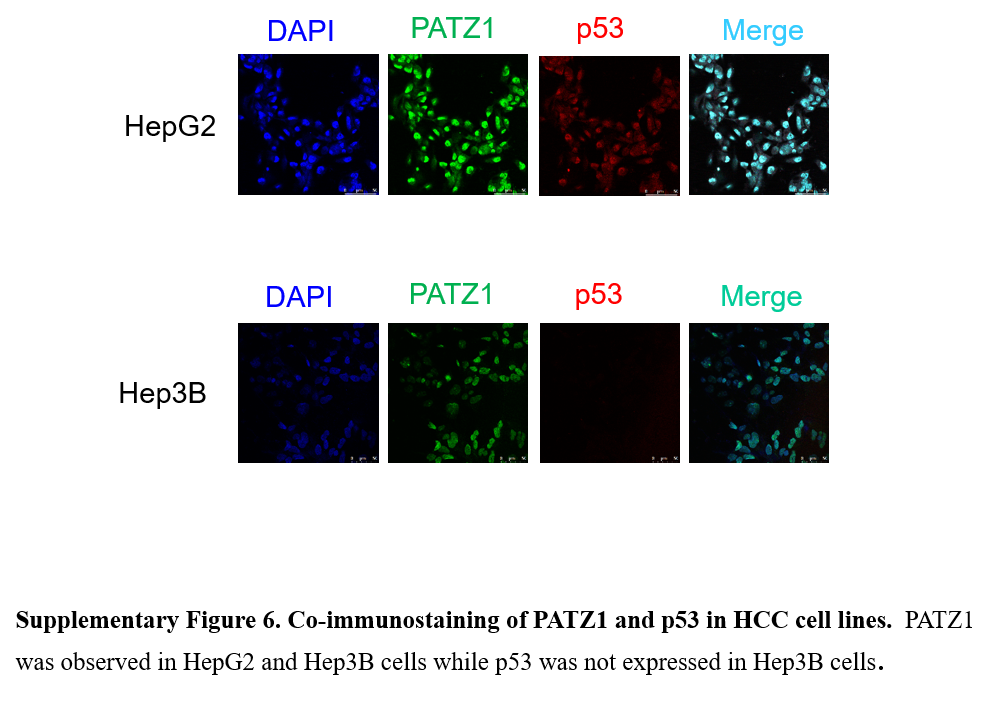

Supplement: Supplementary Figure 6 — Co-immunostaining of PATZ1 and p53 in HCC cell lines. PATZ1 was observed in HepG2 and Hep3B cells while p53 was not expressed in Hep3B cells. [file Image_6.TIF]
