# Supplementary material for: PATZ1 (MAZR) Co-occupies Genomic Sites With p53 and Inhibits Liver Cancer Cell Proliferation via Regulating p27
Source: Front Cell Dev Biol. 2021 Feb 1;9:586150. doi: 10.3389/fcell.2021.586150 (PMC7882738; doi:10.3389/fcell.2021.586150)
Supplement: Supplementary Table 1 — Primers used for qRT-PCR. [file Table_1.DOCX]

**Table S1: Primers used for qRT-PCR**

|  | Forward (5’ 🡪 3’) | Reverse (5’ 🡪 3’) |
| --- | --- | --- |
| *PATZ1* | GGCCTGATCACTTGAACGGA | GGCAGAGGAGAAACCTCGG |
| *CDKN1B* | GGACACGCATTTGGTGGACC | GAATCGTCGGTTGCAGGTCG |
| *CRIP2* | AGCAAAGTCAGGGAGGGTAATG | GAGCTCACCTTCTCGGCTGTT |
| *RAC2* | CGCCAAGTGGTTCCCAGAA | GCACTCCAGGTATTTCACCGA |
| *CCL20* | CGAATCAGAAGCAGCAAGCAA | TTGGATTTGCGCACACAGAC |
| *TBX21* | CAGGGACGGCGGATGTTC | GCCCCCTTGTTGTTTGTGAG |
| *CMPK1* | TCGTCGAGAAATATGGCTACACA | GCCTTCCCATCCATGGTCTT |
| *CAV2* | CGGAGAAGGCGGACGTACA | CCTCGAAGCCCAGCTTGAGAT |
| *CDH2* | ACAGCCGTGTGTGAAAATGC | AGAATTGTTGCCCAGCAGGT |
| *BAX* | GGCCCTTTTGCTTCAGGGTTTCA | CGTCCTGGAGACAGGGACAT |
| *POP5* | TATCAGCTTGTGTGGTCAGC | GACATGTTCTTATTGTACCTCCCA |
| *EIF3F* | AGCACTCTGTGCTGATCCAC | CAGGTCAACTCCGATGCGT |
| *PERP* | CTCATGGAGTACGCGTGGGG | TGGGTGTACTTCACGGGGTA |
| *β-actin* | AGAGCTACGAGCTGCCTGAC | AGCACTGTGTTGGCGTACAG |

**Table S2: Primers used for ChIP-qPCR**

|  | Genomic region | Forward (5’ 🡪 3’) | Reverse (5’ 🡪 3’) |
| --- | --- | --- | --- |
| *CDKN1B* amplicon 1 | CDKN1B TSS -1985/-1654 | TGTTCCTTTTCCCCCAGTCTGCAGT | TGGTACCGGAGTCAGGACTAGATGCAA |
| *CDKN1B* amplicon 2 | CDKN1B TSS -1163/-898 | TGCTCATCGTCCTACTTTACCTTCC | TGCTCCAACAAACTCAGAACAATATTC |
| *CDKN1B* amplicon 3 | CDKN1B TSS -774/-526 | TTAACTGTGCTTGGGAAGGAAGATC | GGCCTAGGGAAGAAGCCAAAG |
| *CDKN1B* amplicon 4 | CDKN1B TSS -307/-70 | ACCTTCGCGGTCCTCTGGT | GATTGGCTGGTCGCGTGAC |
| *CDKN1B* amplicon 5 | CDKN1B TSS +9/+168 | CGCTCGCCAGCCTCGGC | CACCCCGAAAAGACGAGCCCC |
| *CDKN1B* amplicon 6 | CDKN1B TSS +314/+505 | GTCCCCTCTCCTCTCCGCCCTC | GGGCTGCGTAGGGGCGCT |
| *CDKN1B* amplicon 7 | CDKN1B TSS +767/+962 | TCACAAACCCCTAGAGGGCAAG | ACCAAATGCGTGTCCTCAGAGTTA |
| *CDKN1B* amplicon 8 | CDKN1B TSS +1225/+1452 | AAACTGGAGATGGTAAGATCCGATAA | CTCCACCCACAATCCTGGA |
| *p2* | Chr 22 | GGACTCGGAAGAGGTTCACCTTCGG | GTCGCCTCCGCTTGCTGAACTCAATGC |

**Table S3: Top 60 PATZ1 ChIP-Seq targets**

| Chr | Start | End | Fold Enrichment | *P*-value (-log_10_) | Annotation | Distance to TSS | Gene Name |
| --- | --- | --- | --- | --- | --- | --- | --- |
| chr11 | 118306739 | 118307323 | 33.71 | 163.41 | promoter-TSS (NM_005933) | -174 | KMT2A |
| chr9 | 100744993 | 100745585 | 30.93 | 121.68 | promoter-TSS (NM_006401) | -200 | ANP32B |
| chr19 | 18391917 | 18392704 | 28.1 | 132.63 | promoter-TSS (NR_036155) | 155 | JUND |
| chr1 | 211432096 | 211432535 | 26.75 | 99.69 | promoter-TSS (NM_001136223) | -392 | RCOR3 |
| chr8 | 42242795 | 42243318 | 25.72 | 89.15 | Intergenic | -6222 | VDAC3 |
| chr2 | 96011837 | 96012667 | 25.28 | 91.48 | promoter-TSS (NM_001034914) | -516 | KCNIP3 |
| chr16 | 1107478 | 1108215 | 25.26 | 136.86 | Intergenic | -14909 | SSTR5 |
| chr16 | 70472911 | 70473402 | 25.1 | 86.28 | promoter-TSS (NM_006927) | -166 | ST3GAL2 |
| chr12 | 12870778 | 12871298 | 25.08 | 179.72 | exon (NM_004064, exon 1 of 3) | 834 | CDKN1B |
| chr17 | 7787045 | 7787756 | 25.03 | 94.05 | promoter-TSS (NM_001005271) | -722 | CHD3 |
| chr1 | 27560571 | 27561544 | 25 | 156.88 | promoter-TSS (NM_015023) | 51 | WDTC1 |
| chr14 | 100751290 | 100752276 | 24.73 | 86.72 | Intergenic | 8089 | MIR6764 |
| chr22 | 36023687 | 36024213 | 24.67 | 105.22 | Intergenic | -4549 | MB |
| chr9 | 123638447 | 123639385 | 24.07 | 107.03 | intron (NM_001286840, intron 1 of 14) | 690 | PHF19 |
| chr4 | 77870693 | 77871162 | 23.96 | 85.58 | promoter-TSS (NM_018243) | 33 | Sep-11 |
| chr2 | 176957246 | 176957582 | 23.95 | 80.73 | promoter-TSS (NM_000523) | -118 | HOXD13 |
| chr18 | 3448055 | 3448799 | 23.68 | 84.2 | promoter-TSS (NM_001278682) | 843 | TGIF1 |
| chr7 | 4784678 | 4785205 | 23.49 | 84.86 | intron (NM_001037165, intron 2 of 8) | -30320 | AP5Z1 |
| chr20 | 21552466 | 21552983 | 23.13 | 81.44 | intron (NR_109880, intron 1 of 3) | 2063 | LOC101929625 |
| chr17 | 28256463 | 28257040 | 22.88 | 86.75 | promoter-TSS (NM_001145053) | -122 | EFCAB5 |
| chr2 | 43453336 | 43454839 | 22.84 | 90.46 | promoter-TSS (NM_006887) | -262 | LINC01126 |
| chr22 | 50329735 | 50330924 | 22.57 | 85.3 | Intergenic | 18052 | CRELD2 |
| chr5 | 154237373 | 154238081 | 22.41 | 92.54 | promoter-TSS (NM_001301074) | -82 | CNOT8 |
| chr15 | 83735527 | 83736418 | 22.17 | 131.3 | promoter-TSS (NR_039740) | -114 | MIR4515 |
| chr1 | 144519883 | 144520241 | 22.07 | 94.71 | intron (NM_001278267, intron 52 of 130) | 92657 | PFN1P2 |
| chr9 | 131549324 | 131549820 | 21.85 | 72.97 | promoter-TSS (NM_018201) | 86 | TBC1D13 |
| chr17 | 34982217 | 34982706 | 21.64 | 76.34 | Intergenic | 24437 | MRM1 |
| chr19 | 14316240 | 14318524 | 21.52 | 120.36 | promoter-TSS (NM_014921) | -385 | ADGRL1 |
| chr12 | 12871695 | 12871936 | 21.46 | 92.63 | exon (NM_004064, exon 2 of 3) | 1612 | CDKN1B |
| chr2 | 121832564 | 121833087 | 21.37 | 83.27 | Intergenic | 209952 | TFCP2L1 |
| chr1 | 54953744 | 54955433 | 21.35 | 119.02 | Intergenic | -59218 | ACOT11 |
| chr14 | 20811583 | 20811855 | 21.18 | 141.43 | promoter-TSS (NM_005484) | -54 | PARP2 |
| chr20 | 42219002 | 42219709 | 21.02 | 122.74 | promoter-TSS (NM_016004) | -223 | IFT52 |
| chr14 | 54976205 | 54976679 | 20.99 | 67.13 | promoter-TSS (NM_006568) | -145 | CGRRF1 |
| chr10 | 133972948 | 133974088 | 20.91 | 70.65 | intron (NM_001105521, intron 18 of 23) | -26896 | DPYSL4 |
| chr12 | 53773815 | 53774492 | 20.73 | 83.07 | promoter-TSS (NM_003109) | 175 | SP1 |
| chr1 | 205718229 | 205719119 | 20.62 | 69.31 | intron (NM_022731, intron 1 of 6) | 698 | NUCKS1 |
| chr22 | 31988712 | 31989221 | 20.4 | 64.48 | intron (NM_001007467, intron 15 of 32) | 28550 | MIR7109 |
| chr3 | 193851985 | 193853107 | 20.28 | 84.05 | Intergenic | -1385 | HES1 |
| chr1 | 226308990 | 226310243 | 20.25 | 100.1 | Intergenic | 59189 | H3F3AP4 |
| chr19 | 48018348 | 48018945 | 20.21 | 101.82 | promoter-TSS (NM_003827) | -132 | NAPA |
| chr2 | 65827600 | 65828302 | 20.06 | 66.67 | Intergenic | -168295 | SPRED2 |
| chr22 | 38795043 | 38795421 | 20 | 77.27 | promoter-TSS (NR_002821) | -301 | LOC400927 |
| chr19 | 926195 | 927259 | 19.91 | 75.34 | intron (NM_005224, intron 1 of 8) | 690 | ARID3A |
| chr13 | 30424427 | 30425003 | 19.81 | 61.86 | 5' UTR (NM_007106, exon 1 of 5) | 105 | UBL3 |
| chr19 | 4267451 | 4268043 | 19.8 | 79.59 | exon (NM_018074, exon 7 of 8) | -10851 | SHD |
| chr12 | 124395387 | 124397709 | 19.73 | 61.75 | intron (NM_207437, intron 58 of 77) | 60615 | CCDC92 |
| chr1 | 154530994 | 154531594 | 19.69 | 96.18 | promoter-TSS (NM_017582) | -174 | UBE2Q1 |
| chr2 | 132249531 | 132250704 | 19.67 | 66.08 | promoter-TSS (NM_001085365) | -54 | MZT2A |
| chr19 | 50400852 | 50401502 | 19.62 | 80.14 | intron (NR_047577, intron 4 of 10) | -1030 | IL4I1 |
| chr5 | 92918433 | 92918894 | 19.49 | 60.52 | promoter-TSS (NM_005654) | -379 | NR2F1 |
| chr17 | 29035257 | 29037127 | 19.42 | 109.63 | promoter-TSS (NR_024187) | -434 | SUZ12P1 |
| chr6 | 32212628 | 32213163 | 19.32 | 73.44 | Intergenic | -21052 | NOTCH4 |
| chr4 | 184177863 | 184178280 | 19.22 | 59.25 | intron (NM_024949, intron 9 of 22) | 63855 | CLDN22 |
| chr12 | 12869521 | 12870540 | 18.99 | 94.58 | promoter-TSS (NM_004064) | -173 | CDKN1B |
| chr10 | 133974480 | 133975291 | 18.94 | 62.34 | intron (NM_001105521, intron 18 of 23) | -25528 | DPYSL4 |
| chr17 | 7167775 | 7168272 | 18.91 | 69.23 | Intergenic | -1512 | CLDN7 |
| chr18 | 76739516 | 76740042 | 18.72 | 61.13 | promoter-TSS (NM_171999) | -496 | SALL3 |
| chr1 | 22263895 | 22264285 | 18.54 | 74.62 | promoter-TSS (NM_005529) | -300 | HSPG2 |
| chrX | 68505676 | 68506193 | 18.53 | 78.03 | Intergenic | 106535 | LINC00269 |

Table S4: List of HDAC isoforms enriched in PATZ1 ChIP-Seq analysis

| Chr | Start | End | Fold Enrichment | *P*-value (-log_10_) | Annotation | Distance to TSS | Gene Name |
| --- | --- | --- | --- | --- | --- | --- | --- |
| chr17 | 42188538 | 42188957 | 10.78 | 48.3 | intron (NM_001015053, intron 2 of 26) | 12266 | HDAC5 |
| chr6 | 114292204 | 114292777 | 10.43 | 28.47 | promoter-TSS (NR_033441) | -132 | HDAC2 |
| chr17 | 42200726 | 42201502 | 8.64 | 31.45 | promoter-TSS (NM_005474) | -100 | HDAC5 |
| chr7 | 18126175 | 18126700 | 6.13 | 11.14 | promoter-TSS (NM_001204144) | -134 | HDAC9 |
| chr1 | 32757432 | 32757603 | 5.2 | 12 | promoter-TSS (NM_004964) | -190 | HDAC1 |
| chr12 | 48232477 | 48232660 | 4.97 | 8.67 | Intergenic | -18806 | HDAC7 |
| chr3 | 13549307 | 13549491 | 4.92 | 7.88 | Intergenic | 27555 | HDAC11 |
| chr12 | 48212768 | 48213078 | 4.46 | 9.15 | intron (NM_001098416, intron 1 of 24) | 840 | HDAC7 |
| chr12 | 48214531 | 48214655 | 4.44 | 7.2 | promoter-TSS (NM_015401) | -830 | HDAC7 |
| chr12 | 48213970 | 48214098 | 4.24 | 7.19 | promoter-TSS (NM_015401) | -271 | HDAC7 |
| chr17 | 42200023 | 42200255 | 3.88 | 7.42 | intron (NM_001015053, intron 1 of 26) | 875 | HDAC5 |
